# African swine fever incursion risks in Latin America and the Caribbean: informal and legal import pathways

**DOI:** 10.3389/fvets.2025.1587131

**Published:** 2025-04-01

**Authors:** Guillermo Arcega Castillo, Michelle L. Schultze, Rachael Schulte, Rachel A. Schambow, Luis Pablo Hervé-Claude, Emilio A. León, Andres M. Perez

**Affiliations:** ^1^Center for Animal Health and Food Safety, College of Veterinary Medicine, University of Minnesota, St. Paul, MN, United States; ^2^Department of Biomedical Sciences, Ross University School of Veterinary Medicine, Basseterre, Saint Kitts and Nevis

**Keywords:** surveillance gaps, risk assessment, biosecurity enforcement, gray literature, transboundary diseases

## Abstract

African swine fever (ASF) is a devastating hemorrhagic disease of swine with high mortality rates and severe socioeconomic impacts on affected pig industries. In 2021, ASF was reported in the Americas for the first time in 40 years, prompting risk assessments for its introduction and spread. This study evaluates ASF incursion risk across 40 territories in the Caribbean, Central America, North America, and northern South America. A structured, multi-step assessment synthesized peer-reviewed literature, government reports, gray literature, and epidemiological databases to classify two primary ASF incursion pathways: informal imports (e.g., traveler-carried pork, illegal migration, unregulated waste disposal) and legal imports (e.g., trade in live swine and pork products). Territories were categorized as “Probable,” “Unlikely,” or “Unknown,” with certainty levels (Low, Medium, High) based on data robustness. Results indicate ASF incursion is “Probable” (Medium certainty) via informal or formal imports in the Bahamas, British Virgin Islands, Colombia, Cuba, Jamaica, Mexico, Panama, Puerto Rico, Venezuela, Turks and Caicos, and the U.S. Virgin Islands. In contrast, Barbados, Bermuda, Costa Rica, El Salvador, and Guyana were classified as “Unlikely” (Medium certainty) to experience an ASF outbreak under current conditions. Due to insufficient data, 24 territories were categorized as “Unknown” (Low certainty), highlighting critical knowledge gaps. These findings emphasize the need for enhanced surveillance, systematic data-sharing, and regional collaboration to improve risk assessments and implement effective ASF prevention measures in the Americas.

## 1 Introduction

African swine fever (ASF) is a severe hemorrhagic disease with mortality rates reaching 100%, imposing significant socioeconomic burdens on the global pig industry (1–3). Caused by the African swine fever virus (ASFV), a large, enveloped DNA arbovirus, ASF affects only swine, including domestic pigs, wild boar, African warthogs, and other wild suids (4, 5). While ASFV does not infect humans or pose a direct threat to food safety, its high fatality rate among domestic pigs underscores the urgent need for effective preventive and containment measures (6–8). ASF transmission occurs through direct contact between infected and susceptible domestic or wild pigs or infected Ornithodoros ticks, or indirectly via contaminated equipment, fomites, or feed (5, 7). ASFV is stable in the environment—persisting in carcasses and pork products, thereby contaminating disposal sites—and can remain viable for days to years; for instance, it has been documented to survive in frozen meat for more than 2 years and in pig manure for up to 160 days (6, 7, 9–14).

Control is especially challenging due to the lack of an approved vaccine or effective treatment, leading to major disruptions in the global swine sector, as culling of affected herds is a mainstay of control (7, 9). Preventing contact between uninfected and infected pigs, only feeding swill or scraps that have been heated to inactivate the virus, and practicing good biosecurity are essential components of control programs (7, 9). Historical accounts indicate that ASF first reached parts of the Caribbean and South America in the 1970s, particularly affecting Brazil, Cuba, and Hispaniola—the Caribbean island that comprises Haiti and the Dominican Republic—but was fully eliminated by the early 1980s (15–19). Since 2018, ASF has caused widespread outbreaks in Africa, Asia, and Europe, with cases later detected in Hispaniola (20–27). In July 2021, the disease was identified in the Dominican Republic, followed by confirmation in Haiti in September (20, 21, 28). This recurrence on Hispaniola occurred nearly four decades after the region was declared free of ASF (29–31).

Given the Dominican Republic's central role in regional transport, tourism, and trade (32–34), concerns over ASF spreading to other territories in the Americas have risen, prompting risk assessments (34–37). Latin America plays a role in global pork production, supplying nearly 8% of the world's total output (38, 39). The region's swine industry is essential for food security and economic stability, with an estimated five million sows, the majority concentrated in Brazil and Mexico (40). Additionally, wild pig populations are widespread, with their potential distribution covering most of Uruguay, nearly half of Paraguay and Argentina, over a third of Brazil, and a significant portion of Chile (41). This manuscript summarizes and discusses information evaluating ASF spread risk, identifying gaps and needs. By highlighting key vulnerabilities and data gaps, this assessment provides practical recommendations for stakeholders to enhance surveillance, legislation, and cross-border collaboration.

## 2 Methods

This study aimed to evaluate the risk of ASF virus bypassing border inspections through informal and legal import pathways and entering territories across the Caribbean, Central America, North America, and northern South America under current conditions. To achieve this objective, a structured, multi-step risk assessment process was conducted, which included defining clear inclusion criteria, systematically acquiring and synthesizing formal and informal data, categorizing ASF introduction pathways, classifying the likelihood of virus incursion, building expert consensus on classifications, and acknowledging methodological limitations. An overview of the methodological framework and assessment steps is provided in Supplementary Figure 1.

### 2.1 Territory selection

Forty territories were identified as relevant for ASF introduction due to their geographical proximity to Hispaniola. The selected territories, spanning island nations, overseas departments, and mainland jurisdictions in the Caribbean, Central America, North America, and South America, included the Bahamas, Cuba, Venezuela, Colombia, Panama, Barbados, Bermuda, the British Virgin Islands, the Cayman Islands, Costa Rica, Curaçao, Dominica, El Salvador, Guatemala, Guyana, Honduras, Jamaica, Mexico, Nicaragua, Puerto Rico, Saint Martin, St. Vincent and the Grenadines, Suriname, Trinidad and Tobago, the US Virgin Islands, Belize, Anguilla, Antigua and Barbuda, Bonaire, Grenada, Guadeloupe, Martinique, Montserrat, Saba, St. Barthelemy, St. Kitts and Nevis, St. Lucia, Sint Eustatius, Sint Maarten, and the Turks and Caicos Islands.

The rationale for inclusion included:

- Geographic proximity and trade links: Territories sharing maritime routes, air traffic, or land borders with ASF-risk areas.
- Travel volume: High movement of tourists, migrants, or goods that could facilitate disease transmission.
- Historical context: Previous ASF concerns or transboundary animal disease events indicating systemic vulnerabilities.

### 2.2 Data collection and search strategy

To assess ASF introduction risks, sources included peer-reviewed publications, government reports, gray literature, and public databases. A broad search strategy captured information on ASF epidemiology, biosecurity, trade policies, and incursion risks from academic and local sources. Keyword searches were conducted in English and Spanish—such as African swine fever, ASF introduction risk, ASF epidemiology, biosecurity, swine trade, smuggling routes, illegal pork imports, animal disease surveillance, migration, cruise ships, contaminated waste, port inspections, transboundary animal diseases, and veterinary health policies—were conducted to maximize retrieval of pertinent data. For each of these searches, the name of the country under assessment was included to ensure geographically relevant results. Searches were conducted from October 2024 to January 2025.

The search process included direct searches in academic, government, and industry databases, backward and forward citation tracking, searches on official government and international agency platforms, and gray literature inclusion. Peer-reviewed publications were retrieved from PubMed, Scopus, and other scholarly databases. Government reports, including import regulations, disease surveillance updates, and border security guidelines, were retrieved from the websites of ministries of agriculture, customs agencies, and relevant international organizations, such as the Food and Agriculture Organization (FAO) of the United Nations and the World Organization for Animal Health (WOAH). Recognizing that many territories do not publish comprehensive government data or formal risk analyses, news outlets, social media and travel platforms, and conference abstracts were also assessed. Trade databases were also utilized to map pork product flows and identify possible entry points. All citations were systematically documented using Zotero (Corporation for Digital Scholarship, Fairfax, VA, USA) to ensure structured citation management and transparency in the review process.

### 2.3 Classification of ASF introduction pathways

Based on the acquired information, two main pathways were identified through which ASF could enter a given territory, via informal or legal imports of live swine or pork products. Informal imports are non-commercial, unregulated, or semi-covert movement of swine or pork products, including illegal migration. This route included traveler-carried pork (fresh, dried, or processed) packed in luggage, mail parcels, or improperly discarded food waste from cruise liners, cargo ships, and airplanes. Given the mobility of people and consumer goods, small-scale or hidden transport can bypass formal checkpoints. Legal imports are officially documented and regulated routes for trade and humanitarian assistance in live swine or pork commodities, including processed meats, frozen cuts, and other derived products.

### 2.4 Risk categories and certainty levels

Each territory was individually assessed under both pathways and assigned a qualitative risk classification based on the likelihood of ASF incursion based on the sources of information assessed:

- Probable: Evidence suggests that an ASF outbreak may occur given prevailing conditions.
- Unlikely: Evidence suggests that prevailing conditions are not favorable for an ASF incursion.
- Unknown: Data were insufficient, contradictory, or absent, preventing a conclusive assessment.

Because data reliability varied widely, each classification also received a certainty descriptor—Low, Medium, or High—indicating the robustness and clarity of the available evidence. In situations where risk remained ambiguous due to incomplete or conflicting information, additional qualifiers were employed:

- Unknown Suspected Probable: Data are insufficient or contradictory, but available evidence leans toward a probable classification without definitive confirmation.
- Unknown Suspected Unlikely: Data are insufficient or contradictory, but available evidence leans toward an unlikely classification without definitive confirmation.

### 2.5 Consensus and validation

The risk assessment process was conducted by a team of three reviewers. Each reviewer conducted searches for every country using the standardized search strategy. Each reviewer independently assessed retrieved evidence, assigning an initial risk assessment value and certainty level for each pathway and country. The designations and comments were systematically compiled into an Excel (Microsoft, Redmond, WA, USA) database.

If all three reviewers assigned the same risk assessment and certainty level to a country, the classification was finalized by consensus. If discrepancies occurred, a structured discussion was held, where reviewers examined sources, contextual factors, and justifications. Disagreements were resolved by re-evaluating primary sources, including government statements, trade data, and biosecurity policies. Newly released government statements or investigative reports were incorporated if they provided relevant updates affecting the classification.

## 3 Results

**Figure 1:**
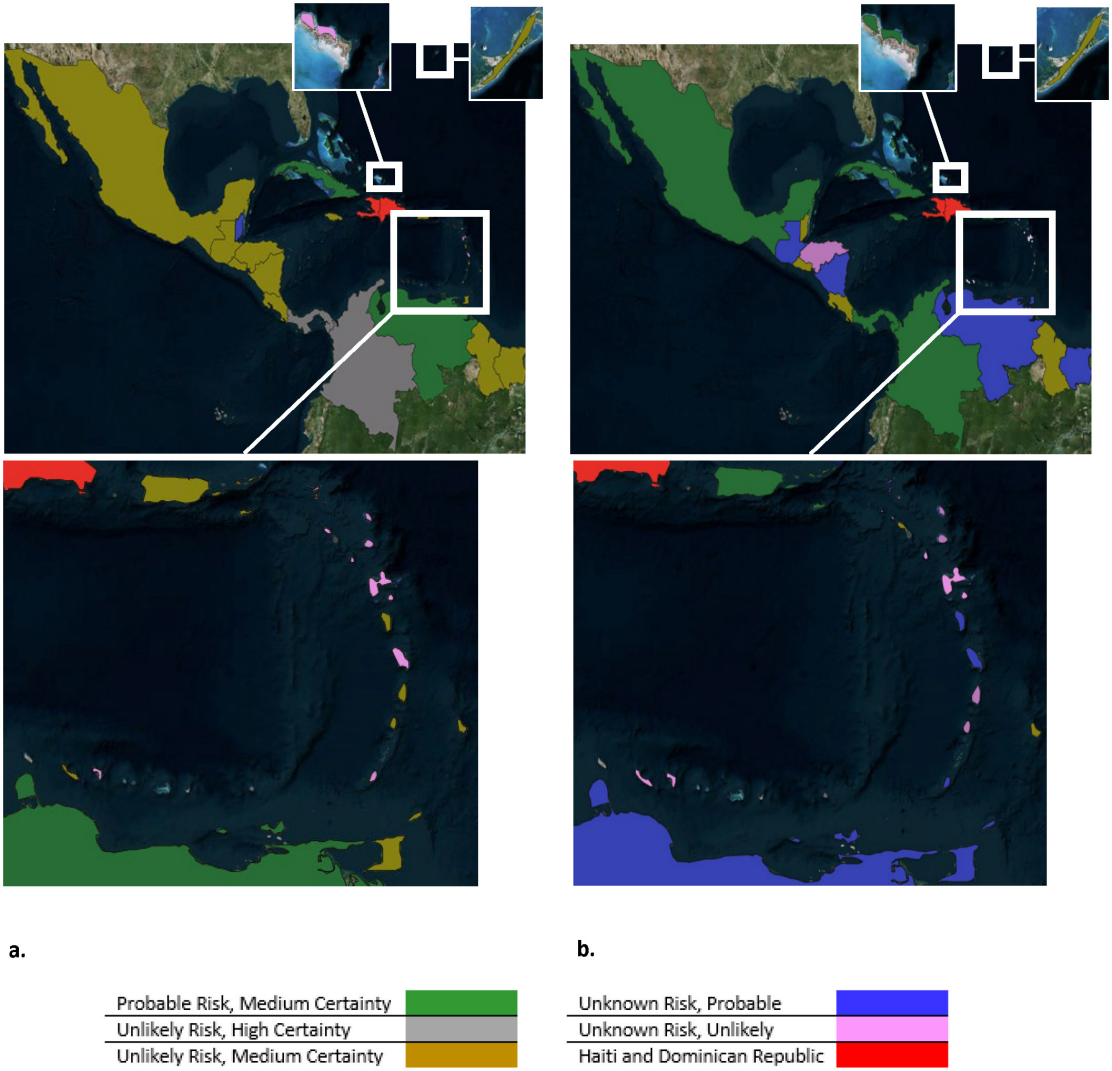
Maps depicted the classification and level of certainty of ASF incursion pathways from **(a)** legal imports and **(b)** informal imports into each territory.

All classification outcomes derived from this assessment are summarized in Table 1, while Figure 1 provides a spatial depiction of ASF risk across the 40 territories. For a detailed breakdown of the assessment results for each country—covering both informal and legal pathways—please refer to Supplementary Tables 1 and 2.

**Table 1:** Classification and level of certainty of ASF incursion pathways from informal imports and legal imports into each territory.

	**Pathways**			
**Territory**	**Informal imports**		**Legal imports**	
	**Level of risk**	**Level of certainty**	**Level of risk**	**Level of certainty**
Anguilla	Unknown, Probable	L	Unknown, Unlikely	L
Antigua and Barbuda	Unknown, Unlikely	L	Unknown, Unlikely	L
Bahamas	Probable	M	Probable	M
Barbados	Unlikely	M	Unlikely	M
Belize	Unlikely	M	Unknown, Probable	L
Bermuda	Unlikely	M	Unlikely	M
Bonaire	Unknown, Unlikely	L	Unknown, Unlikely	L
British Virgin Islands	Probable	M	Unlikely	M
Cayman Islands	Unknown, Unlikely	L	Unlikely	M
Colombia	Probable	M	Unlikely	H
Costa Rica	Unlikely	M	Unlikely	M
Cuba	Probable	M	Probable	M
Curaçao	Unknown, Unlikely	L	Unlikely	M
Dominica	Unknown, Probable	L	Unlikely	M
El Salvador	Unlikely	M	Unlikely	M
Grenada	Unknown, Probable	L	Unknown, Unlikely	L
Guadeloupe	Unknown, Unlikely	L	Unknown, Unlikely	L
Guatemala	Unknown, Probable	L	Unlikely	M
Guyana	Unlikely	M	Unlikely	M
Honduras	Unknown, Unlikely	L	Unlikely	M
Jamaica	Probable	M	Unlikely	M
Martinique	Unknown, Probable	L	Unknown, Unlikely	L
Mexico	Probable	M	Unlikely	M
Montserrat	Unknown, Unlikely	L	Unknown, Unlikely	L
Nicaragua	Unknown, Probable	L	Unlikely	M
Panama	Probable	M	Unlikely	H
Puerto Rico	Probable	M	Unlikely	M
Saba	Unknown, Unlikely	L	Unknown, Unlikely	L
Saint Barthelemy	Unknown, Unlikely	L	Unknown, Unlikely	L
Saint Lucia	Unknown, Unlikely	L	Unlikely	M
Saint Martin	Unknown, Probable	L	Unknown, Unlikely	L
Sint Eustatius	Unknown, Unlikely	L	Unknown, Unlikely	L
Sint Maarten	Unknown, Probable	L	Unknown, Unlikely	L
St. Kitts and Nevis	Unlikely	M	Unknown, Unlikely	L
St. Vincent and the Grenadines	Unknown, Unlikely	L	Unlikely	M
Suriname	Unknown, Probable	L	Unlikely	M
Trinidad and Tobago	Unknown, Probable	L	Unlikely	M
Turks and Caicos	Probable	M	Unknown, Unlikely	L
US Virgin Islands	Probable	M	Unlikely	M
Venezuela	Unknown, Probable	L	Probable	M

### 3.1 Informal imports pathway

The risk of ASF incursion was categorized as “Probable” in 10 territories—the Bahamas, the British Virgin Islands, Colombia, Cuba, Jamaica, Mexico, Panama, Puerto Rico, Turks and Caicos, and the U.S. Virgin Islands—due to lax import regulations (42, 43), porous maritime borders (44), tourism (34, 45), smuggling networks (46–49), cruise ship waste (50), and irregular migrant flows (51–53). Loopholes in import restrictions increase ASF risk, such as Cuba's new regulation allowing vacuum-sealed fresh meat imports (42) and the British Virgin Islands' fluctuating rules on personal food imports (43). Weak maritime enforcement enables smuggling, with Turks and Caicos intercepting 23 migrant vessels in a year, including one carrying 204 individuals (44). Smuggling networks exploit isolated docks and ferry routes, with CBP identifying ferry boats from the Dominican Republic as a high-risk ASF pathway into Puerto Rico and the U.S. Virgin Islands (46, 47).

The Bahamas is a hotspot for human smuggling, with numerous migrants intercepted between 2021 and 2023, primarily from Haiti, the Dominican Republic, and Colombia (48, 49). These routes facilitate unregulated food transport and poor waste management, increasing ASF risks. Similarly, the Dominican Republic, a major tourism hub, is a key ASF introduction pathway, with high visitor numbers from the Bahamas, Colombia, Jamaica, Mexico, Panama, and Puerto Rico (34, 54). Cruise ship waste disposal further threatens biosecurity. Reports show Carnival Corporation illegally dumped 15 pounds of food waste at Half Moon Cay, 500,000 gallons of treated sewage, and plastic-contaminated waste in Bahamian waters (50). Such practices introduce ASF risks via improperly handled waste or scavenging wildlife.

The Darién Gap, a major migration corridor, saw 520,000 crossings in 2023—a 4fold increase from 2021—and 244,000 crossings by September 2024, with Venezuelans making 66% of the total (55). Venezuelans and Haitians have been among the top three nationalities crossing the Darién Gap since 2022, driven by political and economic instability, crime, and the search for better opportunities (53). These mass displacements heighten ASF risks through informal trade, contaminated goods, and unmanaged food waste along migration routes.

Seven territories (Barbados, Belize, Bermuda, Costa Rica, El Salvador, Guyana, and St. Kitts and Nevis) were categorized as “Unlikely,” also with Medium certainty, due to strong enforcement of border regulations (56, 57), regional cooperation with agencies such as FAO and WOAH (58), and strict bans on meat products from ASF-affected regions. In some cases, the risk is further mitigated by the absence of pig populations, such as in Bermuda, where a broadcasting company reported via social media that there were no more pigs in the country as of 2024 (59). Although occasional smuggling may occur, evidence suggests that these territories demonstrated stronger border security measures compared to those in the “Probable” category.

Several territories were categorized as “Unknown” due to insufficient, outdated, or unverified data on infrastructure (60, 61), customs procedures (62), migration patterns (63–68), and unregulated trade (69, 70), all of which are critical factors for ASF risk assessment. Among these, Anguilla, Dominica, Grenada, Guatemala, Martinique, Nicaragua, Saint Martin, Sint Maarten, Suriname, Trinidad and Tobago, and Venezuela were designated as “Unknown Suspected Probable” with Low certainty. This classification reflects a lack of concrete evidence confirming ASF presence while highlighting conditions that could facilitate its introduction and spread. For instance, Guatemala, a key transit country for illicit goods and human migration, seized 5.04 metric tons of cocaine in 2022, yet its porous borders and unregulated trade networks remain largely unmonitored (60). Similarly, Venezuela's ongoing economic and political crisis has displaced over 7.7 million migrants (61, 64), increasing the flow of informal trade and human movement, particularly to Curaçao and Aruba, where migrants reportedly pay up to $500 per crossing (71). In Trinidad and Tobago, an estimated 38,000 Venezuelan refugees and migrants (65) have contributed to growing informal economies and smuggling routes, potentially circumventing official biosecurity controls. Gaps in customs enforcement further facilitate these movements, as seen in places like Grenada, where travelers frequently comment on the ease of bringing food and other goods through customs without rigorous inspection, highlighting broader vulnerabilities in border security across the region (70).

The combined effect of these vulnerabilities introduces significant uncertainty into the ASF risk assessment for these territories. While the conditions for ASF introduction exist, the lack of reliable surveillance, enforcement, and trade documentation prevents a definitive classification, resulting in a Low certainty designation. These findings underscore the need for targeted risk assessments and enhanced monitoring to clarify ASF status and mitigate potential spread in these regions.

In contrast, Antigua and Barbuda, Bonaire, the Cayman Islands, Curaçao, Guadeloupe, Honduras, Montserrat, Saba, Saint Barthelemy, Saint Lucia, Sint Eustatius, and St. Vincent and the Grenadines were labeled “Unknown Suspected Unlikely,” all with Low certainty, as official statements suggested strong border enforcement but lacked sufficient corroboration from independent sources (34, 72–77).

### 3.2 Legal imports pathway

Three territories—Cuba, Venezuela, and the Bahamas—were classified as “Probable” for ASF introduction via legal imports, with Medium certainty. In Cuba, the past importation of pork from ASF-affected regions, such as Russia, raised concerns about potential entry routes (78). Although Cuban authorities validated Russian veterinary control systems (78), enforcement of sanitary measures remains unclear. Venezuela, heavily reliant on large-scale pork imports, has received shipments from ASF-endemic regions, including 13,500 tons of Russian pork in 2020 (79, 80). The lack of transparency in veterinary oversight and limited public reporting on biosecurity measures further exacerbate the risk (80). In the Bahamas, Import Risk Analyses guided import guidelines; importation from ASF affected regions is permitted with veterinary certification of the herd. This oversight reduces risk compared to Cuba and Venezuela, but imports from ASF-affected regions increases risk relative to territories classified as “Unlikely.”

Most other territories—Barbados, Bermuda, British Virgin Islands, Cayman Islands, Costa Rica, Curaçao, Dominica, El Salvador, Guatemala, Guyana, Honduras, Jamaica, Mexico, Nicaragua, Puerto Rico, Saint Lucia, St. Vincent and the Grenadines, Suriname, Trinidad and Tobago, and the U.S. Virgin Islands—were classified as “Unlikely” with Medium certainty. These territories maintain well-documented bans and stringent veterinary controls for pork imports from ASF-positive regions. Some, such as Mexico, Costa Rica, and Jamaica, have less frequent reporting but still demonstrate credible regulatory frameworks (36, 37, 76, 77, 81–93). In contrast, Colombia and Panama were assigned High certainty due to strict ASF screening and biosecurity protocols. Colombia enforces strict border controls to prevent ASF introduction (81). Panama declared a Sanitary Alert, implementing a 16-km biosecurity zone around key ports, alongside quarantine measures for live pig imports (82). Both countries collaborate with international partners (FAO, OIRSA) to strengthen ASF prevention (34, 37).

Some territories lacked transparent data on the existence or enforcement of legal import laws, leading to their classification as “Unknown,” all with Low certainty. Belize received a “Suspected Probable” designation due to partial evidence of weak oversight. While there are risk assessments and import bans on ASF-affected regions, reliance on donor-funded surveillance and the need for heightened containment efforts suggest potential gaps in enforcement (94, 95). Turks and Caicos, Guadeloupe, Anguilla, Antigua and Barbuda, Grenada, Martinique, St. Kitts and Nevis, Saint Martin, Montserrat, Saint Barthelemy, Saba, Sint Eustatius, Bonaire, and Sint Maarten were labeled “Suspected Unlikely” due to official prohibitions. However, limited publicly available data prevented full confirmation of compliance (34, 36, 96–101).

## 4 Discussion

The analysis indicates that there is evidence to suggest that an incursion of ASF—whether through informal or formal import pathways—may be “Probable” in 11 out of the 40 territories studied. These territories—the Bahamas, the British Virgin Islands, Colombia, Cuba, Jamaica, Mexico, Panama, Puerto Rico, Venezuela, Turks and Caicos, and the U.S. Virgin Islands—share overlapping factors previously linked to increased ASF risk in other regions. These include extensive tourism, porous maritime borders, and known smuggling networks, as well as purchasing pork from ASF-affected countries, limited transparency and data sharing with international oversight agencies, and established migration routes that may inadvertently facilitate pathogen movement. These findings align with existing ASF research, which highlights that high-volume human and commodity movement can introduce transboundary animal diseases (102, 103) even under strict legal frameworks (104). Ultimately, weak enforcement of regulations and monitoring gaps at ports, airports, or land crossings remain significant risks for ASF incursion.

In contrast, only 5 out of 40 territories—Barbados, Bermuda, Costa Rica, El Salvador, and Guyana—had evidence and data to conclude that, under prevailing conditions, ASF introduction and subsequent outbreaks are “Unlikely” to occur. These territories have demonstrated relatively robust border-control measures, more transparent veterinary oversight, and consistent adherence to import bans from ASF-affected regions—characteristics that align with international recommendations for preventing disease incursions. By maintaining updated legislation, thorough port-of-entry inspections, and active participation in regional animal health initiatives, these territories show how vigilant policy and practice can mitigate risk when backed by reliable data and transparent reporting (105, 106).

The most critical finding is that for 24 of the 40 territories, available data were insufficient to assess ASF introduction risk accurately. This knowledge gap is a common challenge in animal health risk assessments; without reliable data on border controls, import records, or informal trade and travel pathways, determining true risk is nearly impossible. Key missing information includes unregulated waste disposal, traveler-carried meat volumes, and the effectiveness of local surveillance systems. When documented, data are often scarce or outdated, forcing risk analysis to rely on anecdotal evidence or indirect indicators, reducing certainty. As a result, these territories remain at an “Unknown” risk level, highlighting the need for systematic data collection and reporting to improve ASF risk evaluations in the region.

## 5 Conclusion

Overall, the high proportion of territories with unclear or incomplete data highlights a critical need for enhanced regional collaboration in surveillance, information-sharing, and capacity building. A key recommendation is to establish frameworks—through regional alliances or memoranda of understanding—that promote standardized data collection, transparent reporting, and consistent enforcement of import controls.

The pig farming industry in Latin America is economically significant and critical for regional food security and livelihoods, with substantial implications for both smallholder and commercial operations. Furthermore, extensive wild boar populations in many Latin American countries provide additional reservoirs for ASF, increasing the potential for widespread transmission and environmental persistence. Thus, an ASF introduction would not only disrupt livestock production but also pose significant ecological and economic risks, underscoring the importance of addressing the gaps identified in this study.

Operating with incomplete information is, in reality, a common challenge in animal health risk assessments, and thus, stakeholders must continue to conduct evaluations and implement preventive measures despite data gaps. The goal, however, should be to minimize uncertainty; more comprehensive data, increased transparency, and enhanced cross-border cooperation can improve confidence in risk estimates. Strengthening these endeavors will bolster preparedness and ultimately safeguard both small-scale producers and commercial swine operations against the devastating impacts of ASF. By prioritizing accurate data collection and fostering trust across jurisdictions, the region can strive to achieve the highest possible level of certainty in preventing future ASF incursions.

## Data Availability

The original contributions presented in the study are included in the article/Supplementary material, further inquiries can be directed to the corresponding author.
