# Supplementary material for: African swine fever incursion risks in Latin America and the Caribbean: informal and legal import pathways
Source: Front Vet Sci. 2025 Apr 1;12:1587131. doi: 10.3389/fvets.2025.1587131 (PMC11996821; doi:10.3389/fvets.2025.1587131)
Supplement: Supplementary file 1 [file Table_1.docx]

Supplemental Table 1 : Risk factors and references for targeted territories in the informal imports pathway

|  |  | | |  |  | |
| --- | --- | --- | --- | --- | --- | --- |
| References for all countries: | Organismo Internacional Regional de Sanidad Agropecuaria. Análisis de riesgo sobre la probabilidad de ingreso, establecimiento y diseminación del virus de la peste porcina africana en la porcicultura de los países de la región del OIRSA  [Internet]. 2020 [cited Nov-Dec 2024]. Available from: https://web.oirsa.org/analisis-de-riesgo-sobre-la-probabilidad-de-ingreso-establecimiento-y-diseminacion-del-virus-de-la-peste-porcina-africana-en-la-porcicultura-de-los-paises-de-la-region-del-oirsa/  Rozstalnyy, A., Roche, X., TagoPacheco, D., Kamata, A., BeltranAlcrudo, D., Khomenko, S., Lockhart, C., Urdaz, J., Gioia, G., Gonzalez Serrano, A., VonDobschuetz, S., Dhingra, M., & Sumption, K. 2022. Qualitative risk assessment for African swine fever virus introduction – Caribbean, South, Central and North Americas. FAO Animal Production and Health Papers, No. 186. Rome. <https://doi.org/10.4060/cb8748en> | | | | | |
| Territory | **Level of Risk** | **Level of Certainty** | **Risk Factors** | | | **Sources** |
| Anguilla | Unknown, Probable | L | Anguilla has implemented border controls, a ban on pig imports, and minimal illegal immigration. There are however forum posts of people bringing in spicy sausage, meats and other food products through customs without an issue. In 2024, Anguilla had about 63,500 cruise ship passengers visit, which is one of the lowest number of passengers for all Caribbean stopovers. | | | **Eastern Caribbean Central Bank (ECCB).** Selected tourism statistics [Internet]. [cited 2024 Nov-Dec]. Available from: <https://www.eccb-centralbank.org/statistics-category/external-sector/selected-tourism-statistics/m>  **Ed Hamilton & Co.** Import restrictions [Internet]. [cited 2024 Nov-Dec]. Available from: <https://www.ed-hamilton.com/resources/import-restrictions/>  **Embassies.net.** Anguilla visa for Dominican Republic citizens [Internet]. [cited 2024 Nov-Dec]. Available from: <https://embassies.net/anguilla-visa-for-dominican-republic-citizens>  **Facebook.** [Post about African swine fever in the Caribbean region] [Internet]. [cited 2024 Nov-Dec]. Available from: <https://www.facebook.com/story.php/?story_fbid=1440920912757177&id=168526109996670>  **Moody’s Analytics.** Anguilla net migration [Internet]. [cited 2024 Nov-Dec]. Available from: <https://www.economy.com/anguilla/net-migration>  **The Anguillian.** African swine fever in the Caribbean region [Internet]. 2021 Aug [cited 2024 Nov-Dec]. Available from: <https://theanguillian.com/2021/08/african-swine-fever-in-the-caribbean-region/>  **Tourism Analytics.** Anguilla statistics [Internet]. [cited 2024 Nov-Dec]. Available from: <https://tourismanalytics.com/anguilla-statistics.html>  **TripAdvisor.** Bringing food to Anguilla [Internet]. [cited 2024 Nov-Dec]. Available from: <https://www.tripadvisor.com/ShowTopic-g147238-i388-k14392447-Bringing_food_to_Anguilla-Anguilla.html> |
| Antigua and Barbuda | Unknown, Unlikely | L | Antigua and Barbuda have implemented proactive measures, including enhanced border surveillance and contingency plans, to prevent ASF introduction. Regional collaboration through CAHSFA and national disease response strategies further mitigate risk. The YouTube video depicts people feeding and swimming with pigs, with little evidence of human smuggling. No informal imports are reported to FAO, and migration levels are low. Informal imports are mentioned in online forums but are subject to customs regulations, which prohibit meat imports, as confirmed by customs guidelines. | | | **Antigua Observer.** AB to take precautions against swine fever [Internet]. 2021 Aug 13 [cited 2024 Nov-Dec]. Available from: <https://antiguaobserver.com/ab-to-take-precautions-against-swine-fever/>  **Eastern Caribbean Central Bank (ECCB).** Selected tourism statistics [Internet]. [cited 2024 Nov-Dec]. Available from: <https://www.eccb-centralbank.org/statistics-category/external-sector/selected-tourism-statistics>  **Ed Hamilton & Co.** Import restrictions [Internet]. [cited 2024 Nov-Dec]. Available from: <https://www.ed-hamilton.com/resources/import-restrictions/>  **International Organization for Migration (IOM).** Antigua and Barbuda [Internet]. [cited 2024 Nov-Dec]. Available from: <https://www.iom.int/countries/antigua-and-barbuda>  **Organized Crime Index.** Antigua and Barbuda 2021 [Internet]. 2021 [cited 2024 Nov-Dec]. Available from: <https://ocindex.net/assets/downloads/2021/english/ocindex_profile_antigua_and_barbuda_2021.pdf>  **TripAdvisor.** Bringing food to Antigua [Internet]. 2016 Nov 1 [cited 2024 Nov-Dec]. Available from: <https://www.tripadvisor.co.nz/ShowTopic-g147242-i225-k10006257-Bringing_food_to_antigua-Antigua_Antigua_and_Barbuda.html>  **VisaHQ.** Antigua and Barbuda customs [Internet]. [cited 2024 Nov-Dec]. Available from: <https://www.visahq.com/antigua-barbuda/customs/>  **YouTube.** African swine fever in the Caribbean region [Internet]. 2021 Aug 13 [cited 2024 Nov-Dec]. Available from: <https://www.youtube.com/watch?v=WLQM4FYMBoE&t=195s> |
| Bahamas | Probable | M | The Bahamas has porous maritime borders, known role as a smuggling hub, lax pork import restrictions, and alleged cruise ship dumping. Bahamas allows visitors to bring in frozen or store packaged food (up to 100 pounds) items in their luggage. There are also reports of food being brought in through luggage without being inspected. Carnival Cruise ships admitted to illegally dumping food waste into Half Moon Cay in 2017. There are also reports of Carnival illegally dumping treated sewage and plastic items from their ships into Bahamian waters. The Bahamas also has a long history of being a hub for illegal smuggling of drugs and humans. | | | **Bahamas Agricultural Health and Food Safety Authority (BAHFSA).** Pork imports [Internet]. [cited 2024 Nov-Dec]. Available from: <https://bahfsabahamas.com/sectors/international/pork>  **Bahamas Air Tours.** Pig Beach [Internet]. [cited 2024 Nov-Dec]. Available from: <https://www.bahamasairtours.com/destination/pig-beach/>  **Bahamas Customs Department.** Prohibited and restricted imports and exports [Internet]. [cited 2024 Nov-Dec]. Available from: <https://www.bahamascustoms.gov.bs/imports-and-exports/prohibited-and-restricted-imports-and-exports/>  **Bahama Beach Club.** Questions and answers [Internet]. [cited 2024 Nov-Dec]. Available from: <https://www.bahamabeachclub.com/the-bahamas/questions-and-answers>  **Cruise Law News.** How long will the Bahamas tolerate Carnival trashing its waters and polluting its air? [Internet]. 2019 Aug 27 [cited 2024 Nov-Dec]. Available from: <https://www.cruiselawnews.com/2019/08/articles/pollution/how-long-will-the-bahamas-tolerate-carnival-trashing-its-waters-and-polluting-its-air/>  **Ed Hamilton & Co.** Import restrictions [Internet]. [cited 2024 Nov-Dec]. Available from: <https://www.ed-hamilton.com/resources/import-restrictions/>  **International Organization for Migration (IOM).** Bahamas [Internet]. [cited 2024 Nov-Dec]. Available from: <https://www.iom.int/countries/bahamas>  **Minority Rights Group International.** Haitians [Internet]. [cited 2024 Nov-Dec]. Available from: <https://minorityrights.org/communities/haitians/>  **National Public Radio (NPR).** Carnival Cruise Lines hit with $20 million penalty for environmental crimes [Internet]. 2019 Jun 4 [cited 2024 Nov-Dec]. Available from: <https://www.npr.org/2019/06/04/729622653/carnival-cruise-lines-hit-with-20-million-penalty-for-environmental-crimes>  **The Washington Post.** The deadly sea route from the Bahamas [Internet]. 2023 Mar 23 [cited 2024 Nov-Dec]. Available from: <https://www.washingtonpost.com/nation/interactive/2023/bahamas-human-smuggling-by-boat/>  **TripAdvisor.** Bringing food from USA to Bahamas [Internet]. 2016 Dec 5 [cited 2024 Nov-Dec]. Available from: <https://www.tripadvisor.com/ShowTopic-g147417-i131-k10050629-o60-Bringing_food_from_USA_to_Bahamas-Paradise_Island_New_Providence_Island_Bahamas.html> |
| Barbados | Unlikely | M | Barbados has implemented proactive ASF surveillance programs in collaboration with the USDA and IICA to mitigate the risk of introduction. Despite these efforts, the country faces potential exposure due to its geographic proximity to ASF-affected regions like Hispaniola and the threat of illegal pork imports. Surveillance programs and legislation are still being strengthened. Migration is increasing, with unknown numbers arriving from Haiti. Customs prohibit meat imports. | | | **Barbados Today.** Take heed [Internet]. 2022 Dec 17 [cited 2024 Nov-Dec]. Available from: <https://barbadostoday.bb/2022/12/17/take-heed/>  **Barbados Today.** Pork imports surge ahead of World Cup [Internet]. 2024 Apr 17 [cited 2024 Nov-Dec]. Available from: <https://barbadostoday.bb/2024/04/17/pork-imports-surge-ahead-of-world-cup/>  **Dominica News Online.** Press statement: U.S. Department of Agriculture assists Barbados in African swine fever testing [Internet]. [cited 2024 Nov-Dec]. Available from: <https://dominicanewsonline.com/news/homepage/homepage-carousel/press-statement-u-s-department-of-agriculture-assists-barbados-in-african-swine-fever-testing/>  **Feed Strategy.** Barbados on alert for African swine fever [Internet]. [cited 2024 Nov-Dec]. Available from: <https://www.feedstrategy.com/animal-health-veterinary/african-swine-fever/article/15442410/barbados-on-alert-for-african-swine-fever>  **Inter-American Institute for Cooperation on Agriculture (IICA).** USDA-IICA technical mission to Barbados charts pathway for monitoring and containment of African swine fever [Internet]. [cited 2024 Nov-Dec]. Available from: <https://iica.int/en/press/news/usda-iica-technical-mission-barbados-charts-pathway-monitoring-and-containment-african>  **TripAdvisor.** Bringing food into Barbados [Internet]. 2016 Nov 15 [cited 2024 Nov-Dec]. Available from: <https://www.tripadvisor.com/ShowTopic-g147262-i230-k10028683-Bringing_food_into_Barbados-Barbados.html>  **UNHCR.** Submission by the United Nations High Commissioner for Refugees for the Office of the High Commissioner for Human Rights' Compilation Report – Universal Periodic Review: Barbados [Internet]. 2023 Apr [cited 2024 Nov-Dec]. Available from: <https://upr-info.org/sites/default/files/country-document/2023-04/UNHCR_UPR43_BRB_E_Main.pdf>  **VisaHQ.** Barbados customs [Internet]. [cited 2024 Nov-Dec]. Available from: <https://www.visahq.com/barbados/customs/>  **Visit Barbados.** Important information [Internet]. [cited 2024 Nov-Dec]. Available from: <https://www.visitbarbados.org/plan-your-trip/important-information> |
| Belize | Unlikely | M | Belize is one of Central America’s three main immigrant-receiving countries, although in 2020 the top countries of origin for migrants were ASF-free. Around holidays there is heightened contraband activity leading the Belize Agricultural Health Authority to intensify surveillance operations. Belize has policies related to the destruction and prohibition of wet garbage, there is a lack of information indicating these policies are followed and enforced. There was limited information surrounding the current border policies in place, besides an article from 2013 indicated the modernization of a new Personal Identification and Registration System (PIRS) border management system at all ports of entry. | | | **Belize Agricultural Health Authority (BAHA).** African swine fever [Internet]. [cited 2024 Nov-Dec]. Available from: <https://baha.org.bz/african-swine-fever/>  **Breaking Belize News.** Belize stands to lose $30 million if African swine fever were to attack the pig industry [Internet]. 2021 Aug 5 [cited 2024 Nov-Dec]. Available from: <https://www.breakingbelizenews.com/2021/08/05/belize-stands-to-lose-30-million-if-african-swine-fever-were-to-attack-the-pig-industry/>  **Ed Hamilton & Co.** Import restrictions [Internet]. [cited 2024 Nov-Dec]. Available from: <https://www.ed-hamilton.com/resources/import-restrictions/>  **Government of Belize.** Amnesty background information [Internet]. [cited 2024 Nov-Dec]. Available from: <https://immigration.gov.bz/amnesty-background-information/>  **International Organization for Migration (IOM).** Border management information system goes live in Belize [Internet]. [cited 2024 Nov-Dec]. Available from: <https://www.iom.int/news/border-management-information-system-goes-live-belize>  **Migrants & Refugees Section – Vatican.** Country profile: Belize [Internet]. [cited 2024 Nov-Dec]. Available from: <https://migrants-refugees.va/country-profile/belize/>  **Organismo Internacional Regional de Sanidad Agropecuaria (OIRSA).** African swine fever: Regional preparedness report [Internet]. 2020 Jul 1 [cited 2024 Nov-Dec]. Available from: <https://www.oirsa.org/contenido/2020/AR_PPA_Edici%C3%B3n%20revisada%2001_07_20.pdf>  **San Pedro Sun.** BAHA intensifies surveillance for contraband [Internet]. 2024 Mar 25 [cited 2024 Nov-Dec]. Available from: <https://www.sanpedrosun.com/government/2024/03/25/baha-intensifies-surveillance-for-contraband/>  **U.S. Department of State.** 2022 Country reports on human rights practices: Belize [Internet]. 2022 [cited 2024 Nov-Dec]. Available from: <https://www.state.gov/reports/2022-country-reports-on-human-rights-practices/belize/>  Belize Agricultural Health Authority. African Swine Fever Advisory #1 Belize [Internet]. Belize Agricultural Health Authority; 2019 Mar 6 [cited 2024 Nov-Dec]. Available from: https://baha.org.bz/2019/03/06/african-swine-fever-advisory-1-belize/  Amandala. BAHA issues African Swine Fever advisory [Internet]. Amandala; 2019 Mar 9 [cited 2024 Nov-Dec]. Available from: https://amandala.com.bz/news/baha-issues-african-swine-fever-advisory/ |
| Bermuda | Unlikely | M | No domestic pigs. Must fill out Customs Declaration upon arrival to declare they are bringing in food products. | | | **Facebook.** [Video: 1409021416644079] [Internet]. [cited 2024 Nov-Dec]. Available from: <https://www.facebook.com/watch/?v=1409021416644079>  **TripAdvisor.** Bringing food to Bermuda [Internet]. 2023 Jun 20 [cited 2024 Nov-Dec]. Available from: <https://www.tripadvisor.com/ShowTopic-g147255-i208-k14432680-o10-Bringing_food-Bermuda.html>  **VisaHQ.** Bermuda customs [Internet]. [cited 2024 Nov-Dec]. Available from: <https://www.visahq.com/bermuda/customs/> |
| Bonaire | Unknown, Unlikely | L | Currently a meat ban on meat from the DR due to ASF from forum posts. Unsure if the governmental agencies are enforcing these policies. Are reports of food products, including meat, getting through customs on forums. | | | **Rijksdienst Caribisch Nederland.** Stricter supervision of illegal residence on Bonaire, Saba and St. Eustatius [Internet]. 2023 Jul 6 [cited 2024 Nov-Dec]. Available from: <https://english.rijksdienstcn.com/latest/news/2023/july/06/stricter-supervision-of-illegal-residence-on-bonaire-saba-and-st.-eustatius>  **The Bonaire Reporter.** Import ban on meat from Dominican Republic [Internet]. [cited 2024 Nov-Dec]. Available from: <https://www.facebook.com/TheBonaireReporter/posts/import-ban-on-meat-from-dominican-republicit-is-temporarily-prohibited-to-import/939908186740659/>  **TripAdvisor.** Bringing food into the island [Internet]. 2023 Apr 20 [cited 2024 Nov-Dec]. Available from: <https://www.tripadvisor.com/ShowTopic-g147267-i731-k14365251-Bringing_food_into_the_island-Bonaire.html>  **BES Reporter.** Bonaire Government issues import ban on meat from Dominican Republic [Internet]. [cited 2024 Nov-Dec]. Available from: https://bes-reporter.com/bonaire-government-issues-import-ban-on-meat-from-dominican-republic/ |
| British Virgin Islands | Probable | M | The British Virgin Islands face a likely risk of ASF introduction due to challenges in controlling illegal immigration and informal trade, specifically from Haiti. Meat import regulations require permits for quantities exceeding 18.1 kg (40 lbs), but smaller amounts are often exempt, increasing the risk of informal meat imports. Since 2021, there has been unprecedented growth in the number of cruise ship passengers arriving to BVI, with almost 500,000 passengers arriving between January to August 2023.  There are reports of Haitian migrants being detained, alongside past cases of illegal trade through local ports.  Between 2021-2024, Haitians represent 59% of the total migrants detained, totaling 143 persons.  Haitian immigrants are using the porous borders of the British Virgin Islands, especially unmanned ports to enter the territories. Former officials have been implicated in corruption, raising concerns about customs enforcement. ASF monitoring remains limited, and informal imports of meat not in original packaging have been reported. | | | **British Virgin Islands Government.** Conditions for importation of pigs [Internet]. [cited 2024 Nov-Dec]. Available from: <https://www.bvi.gov.vg/sites/default/files/Conditions%20for%20importation%20of%20pigs.pdf>  **British Virgin Islands Government.** Statement by Honourable Rymer on unprecedented growth in cruise passenger arrivals during summer 2021 [Internet]. 2021 [cited 2024 Nov-Dec]. Available from: <https://www.bvi.gov.vg/media-centre/statement-honourable-rymer-unprecedented-growth-cruise-passenger-arrivals-summer-2021>  **British Virgin Islands Government.** 20 Haitian irregular migrants found on Virgin Gorda [Internet]. [cited 2024 Nov-Dec]. Available from: <https://bvi.gov.vg/media-centre/20-haitian-irregular-migrants-found-virgin-gorda>  **Ed Hamilton & Co.** Import restrictions [Internet]. [cited 2024 Nov-Dec]. Available from: <https://www.ed-hamilton.com/resources/import-restrictions/>  **Financial Investigation Agency British Virgin Islands.** Migrant smuggling: Extent and impact in the Virgin Islands [Internet]. 2024 Oct 3 [cited 2024 Nov-Dec]. Available from: <https://fiabvi.vg/Portals/0/DNNGalleryPro/uploads/2024/10/3/MigrantSmuggling-ExtentandImpactintheVIforPublicUse(FINAL).pdf>  **Loop News.** Former BVI Ports Authority boss sentenced to 9 years for drug scheme [Internet]. [cited 2024 Nov-Dec]. Available from: <https://caribbean.loopnews.com/content/former-bvi-ports-authority-boss-sentenced-9-years-drug-scheme>  **The Aerial BVI.** BVI customs rules: What you can bring [Internet]. [cited 2024 Nov-Dec]. Available from: <https://aerialbvi.com/blog/bvi-customs-rules-what-you-can-bring/>  **TripAdvisor.** Bringing food to the BVI's [Internet]. 2018 [cited 2024 Nov-Dec]. Available from: <https://www.tripadvisor.com/ShowTopic-g147353-i545-k12200265-Bringing_food_to_the_BVI_s-British_Virgin_Islands.html>  **TripAdvisor.** Bringing frozen food/meat [Internet]. 2023 [cited 2024 Nov-Dec]. Available from: <https://www.tripadvisor.com/ShowTopic-g147353-i545-k14371220-Bringing_Frozen_Food_Meat-British_Virgin_Islands.html>  **World Bank.** Net migration, British Virgin Islands [Internet]. [cited 2024 Nov-Dec]. Available from: <https://data.worldbank.org/indicator/SM.POP.NETM?locations=VG> |
| Cayman Islands | Unknown, Unlikely | L | The Cayman has strict import regulations and limited pathways for informal pork importation. However, concerns remain regarding organized human smuggling networks involving Haitian migrants, which could result in ASF introduction. The islands import ships from the Dominican Republic raising additional risks. While regulations restrict informal meat importation, data on migration and the extent of informal imports remain unclear. They have established import protocols but challenges in enforcement and trafficking remains uncertain. | | | **Cayman Islands Department of Agriculture.** Conditions for importation of meat and meat products [Internet]. 2024 Oct [cited 2024 Nov-Dec]. Available from: <https://doa.gov.ky/wp-content/uploads/2024/10/OF-IMP-12-IMPORTATION-OF-MEAT-AND-MEAT-PRODUCTS-Revised-2024.pdf>  **Cayman News Service.** Cayman cops detain Haitian migrants [Internet]. 2018 Mar [cited 2024 Nov-Dec]. Available from: <https://caymannewsservice.com/2018/03/cayman-cops-haitian-migrants/>  **Cayman Islands Port Authority.** Port of origin statistics [Internet]. [cited 2024 Nov-Dec]. Available from: <https://www.caymanport.com/wp-content/uploads/port_of_origin_stats.pdf>  **Cayman Islands Port Authority.** Passenger statistics [Internet]. [cited 2024 Nov-Dec]. Available from: <https://www.caymanport.com/wp-content/uploads/passenger_stats.pdf>  **Cayman Islands Department of Agriculture.** FAQs: Importation of meat and meat products [Internet]. [cited 2024 Nov-Dec]. Available from: <https://doa.gov.ky/faqs/#:~:text=Individual%20traveling%20passengers%20are%20allowed,bears%20the%20inspection%20mark%2Ficon> |
| Colombia | Probable | M | Colombia has a strong surveillance but vast vulnerabilities in migration routes. Rigorous controls include 100% inspections at ports, airports, and borders, coupled with physical inspections of high-risk shipments and diagnostic testing in areas like Córdoba. However, the country’s role as a major transit point for migrants through the Darién Gap elevates risk of ASF introduction.  All migrants crossing the Darién Gap must pass through Colombia, with many arriving through its porous border with Venezuela. Although customs efforts include prohibitions on pork products for human consumption, there is incomplete enforcement along with reports of informal imports. In 2018, Colombia was a top travel destination for Dominican Republicans, with 25,631 people traveling from the DR. | | | **3tres3.com.** México y Colombia toman medidas ante la PPA en República Dominicana [Internet]. [cited 2024 Nov-Dec]. Available from: <https://www.3tres3.com/latam/ultima-hora/mexico-y-colombia-toman-medidas-ante-la-ppa-en-republica-dominicana_13375/>  **Agrosavia.** [Internet]. [cited 2024 Nov-Dec]. Available from: <https://repository.agrosavia.co/handle/20.500.12324/23254>  **Migration Policy Institute.** Haitian migration through the Americas [Internet]. [cited 2024 Nov-Dec]. Available from: <https://www.migrationpolicy.org/article/haitian-migration-through-americas>  **3tres3.com.** Colombia extrema las medidas de vigilancia de la peste porcina africana [Internet]. [cited 2024 Nov-Dec]. Available from: <https://www.3tres3.com/latam/ultima-hora/colombia-extrema-las-medidas-de-vigilancia-de-la-peste-porcina-african_13444/>  **Migration Policy Institute.** Haiti: Next displacement crisis in the Americas? [Internet]. [cited 2024 Nov-Dec]. Available from: <https://www.migrationpolicy.org/news/haiti-next-displacement-crisis-americas>  **Refugees International.** After the Darién: Aid and pathways for migrants in Panama and Costa Rica [Internet]. [cited 2024 Nov-Dec]. Available from: <https://www.refugeesinternational.org/reports-briefs/after-the-darien-aid-and-pathways-for-migrants-in-panama-and-costa-rica/>  **Pig333.com.** Colombia establishes health measures to prevent ASF [Internet]. [cited 2024 Nov-Dec]. Available from: <https://www.pig333.com/latest_swine_news/colombia-establishes-health-measures-to-prevent-asf_15825/>  **Pig333.com.** Colombia activates measures to avoid the entrance of ASF [Internet]. [cited 2024 Nov-Dec]. Available from: <https://www.pig333.com/latest_swine_news/colombia-activates-measures-to-avoid-the-entrance-of-asf_14949/>  **Council on Foreign Relations.** Crossing the Darién Gap: Migrants risk death on the journey to the U.S. [Internet]. [cited 2024 Nov-Dec]. Available from: <https://www.cfr.org/article/crossing-darien-gap-migrants-risk-death-journey-us>  **Washington Office on Latin America (WOLA).** Migrants in Colombia: Between government absence and criminal control [Internet]. [cited 2024 Nov-Dec]. Available from: <https://www.wola.org/analysis/migrants-in-colombia-between-government-absence-and-criminal-control/#_Toc169245452>  **TripAdvisor.** Taking food to Colombia [Internet]. [cited 2024 Nov-Dec]. Available from: <https://www.tripadvisor.com/ShowTopic-g294073-i1708-k14232481-Taking_food_to_Colombia-Colombia.html>  **3tres3.com.** Colombia extrema las medidas de prevención contra la PPA [Internet]. [cited 2024 Nov-Dec]. Available from: <https://www.3tres3.com/es-ar/ultima-hora/colombia-extrema-las-medidas-de-prevencion-contra-la-ppa_13382/>  **YouTube.** [Internet]. [cited 2024 Nov-Dec]. Available from: <https://www.youtube.com/watch?v=d-9wAWTL6aU> |
| Costa Rica | Unlikely | M | Costa Rica is a transit country for migrants. While monitoring efforts focus primarily on classical swine fever, ASF surveillance remains passive, and inconsistent documentation at entry points. The country buses migrants after they cross the Darién Gap and is more welcoming to migrants than other nations. A 2020 report highlighted a porous border with Nicaragua, though most crossings involved Nicaraguans fleeing economic conditions. Informal pork imports occur from non-ASF-affected areas, with small amounts confiscated. While policies restrict pork transportation, some reports suggest smuggling remains possible. High numbers of Latin American migrants, including those working in agriculture are present in the territory. | | | **El Faro.** Migrant trafficking on the Nicaragua–Costa Rica border [Internet]. 2020 Dec. Available from: <https://elfaro.net/en/202012/centroamerica/0000025075-migrant-trafficking-on-the-nicaragua-ndash-costa-rica-border?tpl=11&u=st-full_text%3Dall&utm_campaign=text-0000025075_migrant-trafficking-on-the-nicaragua-ndash-costa-rica-border&utm_medium=initdocu&utm>  **Miami Herald.** Venezuela-related news article [Internet]. 2023 Sep. Available from: <https://www.miamiherald.com/news/nation-world/world/americas/venezuela/article279839044.html>  **Migration Data Portal.** National migration data for Costa Rica [Internet]. Available from: <https://www.migrationdataportal.org/es/dashboard/national-data?c=188&i=10681&t=2010>  **Migration Policy Institute.** Costa Rica: Nicaragua migrants face subtle barriers [Internet]. Available from: <https://www.migrationpolicy.org/article/costa-rica-nicaragua-migrants-subtle-barriers>  **Migration Policy Institute.** Haitian migration through the Americas [Internet]. Available from: <https://www.migrationpolicy.org/article/haitian-migration-through-americas>  **Migrants & Refugees Section (Vatican).** Country profile: Costa Rica [Internet]. Available from: <https://migrants-refugees.va/country-profile/costa-rica/>  **Reddit - Costa Rica Travel.** Can I bring packaged food into Costa Rica? [Internet]. Available from: <https://www.reddit.com/r/CostaRicaTravel/comments/1ds360d/can_i_bring_packaged_food_into_costa_rica/>  **Refugees International.** After the Darién: Aid and pathways for migrants in Panama and Costa Rica [Internet]. Available from: <https://www.refugeesinternational.org/reports-briefs/after-the-darien-aid-and-pathways-for-migrants-in-panama-and-costa-rica/> |
| Cuba | Probable | M | Cuba has a likely risk of ASF introduction due to its reliance on informal food imports, including pork products, and challenges in enforcing strict biosecurity measures at its borders. While customs controls exist to prevent the entry of unprocessed pork products, the reliance on imports (80% of the food Cubans consume is imported) and the food shortage increases risk. As of 2022 restrictions on personal imports of food were relaxed which allowed vacuum packed fresh meat, sausages, and other foods from certain ASF-free countries as a means to help alleviate the food shortage. There are reports of customs seizing illegal pork products.  Proximity to Haiti and the Dominican Republic greatly increases this risk, with significant numbers of travelers between these regions (15,000 to Haiti in 2019 and 60,000 to the DR in 2021) and migrants from these regions. Migrants attempting to reach US or other destinations may go through Cuba, further complicating biosecurity efforts. | | | Associated Press. Boat of 842 Haitian migrants lands in Cuba [Internet]. 2022. [cited Nov-Dec 2024]. Available from: <https://www.ap.org/news-highlights/best-of-the-week/2022/boat-of-842-haitian-migrants-lands-in-cuba/>.  Cibercuba. Cuba establishes new food import regulations [Internet]. 2024 Jan 17. [cited Nov-Dec 2024]. Available from: <https://en.cibercuba.com/noticias/2024-01-17-u1-e129488-s27061-cuba-establece-nuevas-regulaciones-importacion-alimentos>.  Directorio Cubano. Cuba seizes pork-based food at customs [Internet]. [cited Nov-Dec 2024]. Available from: <https://www.directoriocubano.info/regulaciones-aduanales/cuba-decomisa-alimentos-de-origen-porcino-en-aduana/>.  EnAlimentos. Russian companies approved to export poultry, pork, and beef to Cuba [Internet]. [cited Nov-Dec 2024]. Available from: <https://enalimentos.lat/noticias/10084-empresas-rusas-podran-vender-carne-de-pollo-cerdo-y-vacuno-a-cuba.html>.  International Organization for Migration. Country profile: Cuba [Internet]. [cited Nov-Dec 2024]. Available from: <https://www.iom.int/countries/cuba>.  OnCuba News. What foods can be brought to Cuba, and what imports are forbidden? [Internet]. [cited Nov-Dec 2024]. Available from: <https://oncubanews.com/en/cuba/what-foods-can-be-brought-to-cuba-and-what-imports-are-forbidden/>.  Reddit - Cuba. Taking food/meat to Cuba [Internet]. [cited Nov-Dec 2024]. Available from: <https://www.reddit.com/r/cuba/comments/131bi9u/taking_food_meat_to_cuba/>.  Translating Cuba. Cubans will be able to import raw meat and other vacuum-packed foods [Internet]. [cited Nov-Dec 2024]. Available from: <https://translatingcuba.com/cubans-will-be-able-to-import-raw-meat-and-other-vacuum-packed-foods/>.  TripAdvisor. What food products are allowed to bring into Cuba? [Internet]. [cited Nov-Dec 2024]. Available from: <https://www.tripadvisor.com/ShowTopic-g147270-i91-k14609084-What_food_products_allowed_to_bring_into_Cuba-Cuba.html>. |
| Curaçao | Unknown, Unlikely | L | Curaçao enforces import bans on meat products from. They have porous borders with inconsistent enforcement that contribute to ongoing smuggling. Reports indicate informal imports bypass customs in Curaçao and Bonaire, though meat is not explicitly mentioned in their customs regulations. | | | CBS Open Data. Population Caribbean Netherlands [Internet]. [cited Nov-Dec 2024]. Available from: <https://opendata.cbs.nl/statline/#/CBS/en/dataset/85007ENG/table?dl=5FC21>.  Tourism Analytics. Caribbean tourism statistics [Internet]. [cited Nov-Dec 2024]. Available from: <https://tourismanalytics.com/caribbean-statistics.html>. |
| Dominica | Unknown, Probable | L | Dominica has partial documentation of importation practices and enforcement of surveillance protocols. There is documentation of law enforcement undergoing training in 2024 to improve immigration and border control activities. There are regulations for travelers in place with pork imports and all meat imports requiring Phytosanitary Certificate and Import Permits. Due to the significant point of transit of Haitian migrants through Dominica en route to Guadeloupe or Martinique, the International Organization for Migration (IOM) started a project to improve the weak immigration infrastructure and border management; however, high numbers of migration through Dominica increase risk to likely. | | | ECCB Central Bank. Selected tourism statistics [Internet]. [cited Nov-Dec 2024]. Available from: <https://www.eccb-centralbank.org/statistics-category/external-sector/selected-tourism-statistics>.  UNDP. Migration in the Dominican Republic: context, challenges, and opportunities [Internet]. [cited Nov-Dec 2024]. Available from: <https://www.undp.org/latin-america/publications/migration-dominican-republic-context-challenges-and-opportunities>.  Dominica Customs. Prohibited and restricted items [Internet]. [cited Nov-Dec 2024]. Available from: <https://customs.gov.dm/index.php/trade/prohibited-and-restricted-items#:~:text=Importation%20of%20meat%20must%20be,from%20the%20Division%20of%20Agriculture>.  IOM. Dominica law enforcement personnel undergo IOM ToT on border management [Internet]. [cited Nov-Dec 2024]. Available from: <https://www.iom.int/news/dominica-law-enforcement-personnel-undergo-iom-tot-border-management>.  IOM. Enhancing Dominica’s border management capacity [Internet]. [cited Nov-Dec 2024]. Available from: <https://www.iom.int/project/enhancing-dominicas-border-management-capacity#:~:text=Dominica%20is%20known%20to%20be%20a%20point,migrants%20>. |
| El Salvador | Unlikely | M | El Salvador has a low risk of ASF introduction due to preventive measures such as airport quarantine, canine detection units, and disinfection protocols. While border inspections aim to limit ASF introduction, ongoing illegal activity and undocumented enforcement measures reduce confidence in risk mitigation. Haitian migration is not a primary concern, as most migrants left Haiti after the 2010 earthquake. Informal importation of pork products is primarily from Guatemala, but there is no specific information on restrictions of animal products in personal luggage. The use of canine units at airports provides an additional layer of protection. | | | OIRSA. African Swine Fever Revised Edition [Internet]. [cited Nov-Dec 2024]. Available from: <https://www.oirsa.org/contenido/2020/AR_PPA_Edici%C3%B3n%20revisada%2001_07_20.pdf>.  Government of Mexico. El Salvador Country Report [Internet]. [cited Nov-Dec 2024]. Available from: <https://www.gob.mx/cms/uploads/attachment/file/785147/El_Salvador_ENG.pdf>.  OIRSA. El Salvador strengthens prevention measures against African Swine Fever (ASF) [Internet]. [cited Nov-Dec 2024]. Available from: <https://web.oirsa.org/en/el-salvador-fortalece-medidas-de-prevencion-ante-peste-porcina-africana-ppa/>.  MAG. Agreement between MAG and OIRSA for canine units and solid waste treatment [Internet]. [cited Nov-Dec 2024]. Available from: <https://www.mag.gob.sv/programas/convenio-mag-oirsa-para-unidades-caninas-y-tratamientos-de-desechos-solidos-aereos/>.  Migration Policy Institute. Haitian migration through the Americas [Internet]. [cited Nov-Dec 2024]. Available from: <https://www.migrationpolicy.org/article/haitian-migration-through-americas>.  Humanitarian Action. Presence and response plans [Internet]. [cited Nov-Dec 2024]. Available from: <https://humanitarianaction.info/plan/1172/presence>. |
| Grenada | Unknown, Probable | L | In the summer of 2024, Hurricane Beryl caused significant damage to Grenada’s infrastructure, power, communications and logistics (90). Within the islands of Carriacou and Petit Martinique, 70% and 97% of buildings respectively were damaged or destroyed. Haiti and Grenada are both members of the Caribbean Community (Caricom) grouping, which means there are limited grounds to prevent a Caricom national from entering a member country. In 2023, Grenada deported 15 Haitians based on these limited grounds on the amount of cash they were carrying, which prevented them from being able to stay in an accommodation for one night. Limited documentation on informal pork imports or prohibition of meat products besides forum posts advising declaration of food items. | | | Loop News Caribbean. Grenada to deport 15 Haitian nationals [Internet]. [cited Nov-Dec 2024]. Available from: <https://caribbean.loopnews.com/content/grenada-deport-15-haitian-nationals>.  Eastern Caribbean Central Bank. Selected tourism statistics [Internet]. [cited Nov-Dec 2024]. Available from: <https://www.eccb-centralbank.org/statistics-category/external-sector/selected-tourism-statistics>.  United Nations Office for the Coordination of Humanitarian Affairs. After the storm: Two months after Hurricane Beryl [Internet]. [cited Nov-Dec 2024]. Available from: <https://www.unocha.org/news/after-storm-two-months-after-hurricane-beryl>.  TripAdvisor. Bringing food items through customs - Grenada [Internet]. [cited Nov-Dec 2024]. Available from: <https://www.tripadvisor.com/ShowTopic-g147295-i518-k14327588-Bringing_food_items_thru_customs-Grenada.html>.  Ed Hamilton & Co. Import restrictions [Internet]. [cited Nov-Dec 2024]. Available from: <https://www.ed-hamilton.com/resources/import-restrictions/>. |
| Guadeloupe | Unknown, Unlikely | L | Guadeloupe has a strong cultural connection to Haiti, but most Haitians who enter illegally do so through other territories (such as Dominica) first. All goods that enter the territory are subject to being checked at the border. Guadeloupe has built strong local research communities and surveillance networks to prevent and control infectious diseases, although there is a lack of specific biosecurity measures pertaining to ASF. They do allow frozen meat imports for personal use in limited quantities and only with a USDA approved label.  Its strong travel ties increase exposure, while biosecurity documentation gaps add uncertainty. | | | Frontiers in Public Health. [Internet]. [cited Nov-Dec 2024]. Available from: <https://www.frontiersin.org/journals/public-health/articles/10.3389/fpubh.2021.652079/full>.  VisaHQ. Guadeloupe customs [Internet]. [cited Nov-Dec 2024]. Available from: <https://www.visahq.com/guadeloupe/customs/>.  Direction de l'Alimentation, de l'Agriculture et de la Forêt de Guadeloupe. L'État agit pour vous : La protection du territoire - Contrôle DAAF/Douane [Internet]. [cited Nov-Dec 2024]. Available from: <https://daaf.guadeloupe.agriculture.gouv.fr/l-etat-agit-pour-vous-la-protection-du-territoire-controle-daaf-douane-a-l-a1650.html>.  TripAdvisor. Bringing food - Guadeloupe [Internet]. [cited Nov-Dec 2024]. Available from: <https://www.tripadvisor.com/ShowTopic-g147300-i1141-k14607815-Bringing_food-Guadeloupe.html>.  Reddit. Thoughts of Martiniquans [Internet]. [cited Nov-Dec 2024]. Available from: <https://www.reddit.com/r/haiti/comments/z14hf4/thoughts_of_martiniquans/?rdt=59814>.  Ed Hamilton & Co. Import restrictions [Internet]. [cited Nov-Dec 2024]. Available from: <https://www.ed-hamilton.com/resources/import-restrictions/>.  Le Monde. En Guadeloupe, les associations dénoncent la gestion des migrants haïtiens par les autorités [Internet]. [cited Nov-Dec 2024]. Available from: <https://www.lemonde.fr/politique/article/2024/12/19/en-guadeloupe-les-associations-denoncent-la-gestion-des-migrants-haitiens-par-les-autorites_6457215_823448.html>. |
| Guatemala | Unknown, Probable | L | Guatemala has records of illegal imports, travel, and immigration including Haiti. Border controls, enhanced inspections, and canine detection units help mitigate risk, alongside outreach campaigns and epidemiologist training. However, there is limited data on travel patterns, irregular migration, and enforcement effectiveness at porous land borders. The eastern border with Belize is problematic due to weak state presence, corruption, and cartel activity, facilitating contraband smuggling, though the extent of pork product trafficking is unclear. Moderate amounts of informal imports have been seized, including meat from the Dominican Republic, with a higher risk linked to informal airport and land border imports. | | | OIRSA. Edición revisada sobre peste porcina africana [Internet]. [cited Nov-Dec 2024]. Available from: <https://www.oirsa.org/contenido/2020/AR_PPA_Edici%C3%B3n%20revisada%2001_07_20.pdf>.  OIRSA. Seminario regional sobre peste porcina africana se desarrolla en Guatemala [Internet]. [cited Nov-Dec 2024]. Available from: <https://web.oirsa.org/seminario-regional-sobre-peste-porcina-africana-se-desarrolla-en-guatemala/>.  SwissInfo. Guatemala endurece vigilancia fronteriza para evitar ingreso de peste porcina [Internet]. [cited Nov-Dec 2024]. Available from: <https://www.swissinfo.ch/spa/guatemala-endurece-vigilancia-fronteriza-para-evitar-ingreso-de-peste-porcina/47260712>.  Pig333. Guatemala and FAO unite to prevent African swine fever [Internet]. [cited Nov-Dec 2024]. Available from: <https://www.pig333.com/latest_swine_news/guatemala-and-fao-unite-to-prevent-african-swine-fever_19588/>.  Prensa Libre. Guatemala prevé aumento de migrantes irregulares haitianos por la crisis que viven [Internet]. [cited Nov-Dec 2024]. Available from: <https://www.prensalibre.com/guatemala/migrantes/guatemala-preve-aumento-de-migrantes-irregulares-haitianos-por-la-crisis-que-viven/>.  IOM. Migración en tránsito [Internet]. [cited Nov-Dec 2024]. Available from: <https://infounitnca.iom.int/migracion-en-transito/>.  FIU Gordon Institute. Guatemala’s security challenges and the government’s response [Internet]. [cited Nov-Dec 2024]. Available from: <https://gordoninstitute.fiu.edu/news-events/the-policy-spotlight/2024/guatemalas-security-challenges-and-the-governments-response.html>.  Migration Policy Institute. Haitian migration through the Americas [Internet]. [cited Nov-Dec 2024]. Available from: <https://www.migrationpolicy.org/article/haitian-migration-through-americas>.  VisaHQ. Guatemala customs [Internet]. [cited Nov-Dec 2024]. Available from: <https://www.visahq.com/guatemala/customs/>. |
| Guyana | Unlikely | M | Guyana has implemented preventive measures, including banning pork imports from affected regions, enhanced surveillance by the GLDA, and strict quarantine protocols for travelers. Guyana has also increased monitoring of flights from the Dominican Republic since 2021. While smuggling activities involving Haitian migrants and porous borders present some challenges, restrictions on informal imports of fresh or raw meat and limited reported cases of illegal pork entry reinforce the country’s biosecurity efforts. Although migration from Venezuela and Haiti is increasing, the overall risk remains low. | | | Breaking Belize News. Central America, Belize stepping up defense against African swine fever [Internet]. 2021 Oct 19 [cited 2024 Nov-Dec]. Available from: <https://www.breakingbelizenews.com/2021/10/19/central-america-belize-stepping-up-defense-against-africa-swine-fever/>  Migration Policy Institute. Haitian migration through the Americas [Internet]. 2024. [cited 2024 Nov-Dec]. Available from: <https://www.migrationpolicy.org/article/haitian-migration-through-americas>  Migration Policy Institute. Guyana’s discovery of oil, labor migration, and climate displacement [Internet]. 2024. [cited 2024 Nov-Dec]. Available from: <https://www.migrationpolicy.org/article/guyana-discovery-oil-labor-migration-climate-displacement#:~:text=Given%20Guyana's%20recent%20history%20as,of%20the%20country's%20overall%20population>.  VisaHQ. Guyana customs regulations [Internet]. 2024. [cited 2024 Nov-Dec]. Available from: <https://www.visahq.com/guyana/customs/> |
| Honduras | Unknown, Unlikely | L | Honduras has strong preventive measures, including field simulations, border inspections, and quarantine systems at ports, airports, and land crossings. Training programs for veterinarians and field technicians by SENASA further enhance early detection capabilities. While the country is a key transit point for migrants, including those from ASF-affected regions, improved migrant registration and tracking, along with low reported seizures of informal meat imports, reduce the likelihood of ASF introduction. Lessons learned from a prior ASF-related border closure in 2010 have informed Honduras’s current strategies, reinforcing its commitment to biosecurity. | | | OIRSA. Edición revisada sobre peste porcina africana [Internet]. [cited Nov-Dec 2024]. Available from: <https://www.oirsa.org/contenido/2020/AR_PPA_Edici%C3%B3n%20revisada%2001_07_20.pdf>.  IOM. Migración en tránsito [Internet]. [cited Nov-Dec 2024]. Available from: <https://infounitnca.iom.int/migracion-en-transito/>.  PorciNews. Honduras: capacitación para evitar la peste porcina africana [Internet]. [cited Nov-Dec 2024]. Available from: <https://porcinews.com/honduras-capacitacion-para-evitar-la-peste-porcina-africana/>.  La Tribuna. Honduras crea cerco contra la peste porcina africana [Internet]. [cited Nov-Dec 2024]. Available from: <https://www.latribuna.hn/2024/09/04/honduras-crea-cerco-contra-la-peste-porcina-africana/>.  Portal Veterinaria. Honduras cierra frontera a carne de cerdo de Guatemala por un brote de peste [Internet]. [cited Nov-Dec 2024]. Available from: <https://www.portalveterinaria.com/actualidad-veterinaria/actualidad/6691/honduras-cierra-frontera-a-carne-de-cerdo-de-guatemala-por-un-brote-de-peste.html>.  WOLA. Halfway to the US: Report on Honduras migration [Internet]. [cited Nov-Dec 2024]. Available from: <https://www.wola.org/analysis/halfway-to-us-report-honduras-migration/#migrantstransiting>.  Migration Policy Institute. Haitian migration through the Americas [Internet]. [cited Nov-Dec 2024]. Available from: <https://www.migrationpolicy.org/article/haitian-migration-through-americas>.  WOLA. Halfway to the US: Report on Honduras migration [Internet]. [cited Nov-Dec 2024]. Available from: <https://www.wola.org/analysis/halfway-to-us-report-honduras-migration/>.  Humanitarian Action. Honduras: Presence and response [Internet]. [cited Nov-Dec 2024]. Available from: <https://humanitarianaction.info/plan/1174/presence>.  VisaHQ. Honduras customs [Internet]. [cited Nov-Dec 2024]. Available from: <https://www.visahq.com/honduras/customs/>. |
| Jamaica | Probable | M | Jamaica has enacted enhanced border surveillance, diagnostic readiness, and collaborative biosecurity initiatives as a result of ASF in the surrounding areas. While there is government coordination to reduce smuggling and illegal migration, the proximity of Jamaica to Haiti and the DR makes the risk of ASF introduction likely through informal imports. Migrants from Haiti often arrive in Jamaica. Illegal pork imports are suspected, and trafficking of non-perishable food items, including potential ASF carriers like beef jerky, poses additional challenges. The agriculture ministry has prioritized industry protection in 2024, with customs enforcing strict controls on meat imports. Small amounts of informal imports from the U.S. and Europe have been seized. | | | Jamaica Information Service. High alert for prevention of African swine fever [Internet]. 2024 [cited Nov-Dec 2024]. Available from: <https://jis.gov.jm/features/high-alert-for-prevention-of-african-swine-fever/>.  Ministry of Agriculture, Jamaica. Agriculture Ministry now able to investigate and diagnose African swine fever [Internet]. 2024 [cited Nov-Dec 2024]. Available from: <https://www.moa.gov.jm/content/agriculture-ministry-now-able-investigate-and-diagnose-african-swine-fever>.  The Guardian. Human rights groups want Jamaica to suspend deportation of illegal Haitian migrants [Internet]. 2024 Sep 10 [cited Nov-Dec 2024]. Available from: <https://www.guardian.co.tt/news/human-rights-groups-want-jamaica-to-suspend-deportation-of-illegal-haitian-migrants-6.2.2129990.d1b77f5200>.  YouTube. Arrival of Haitian migrants in Jamaica [Internet]. 2024 [cited Nov-Dec 2024]. Available from: <https://www.youtube.com/watch?v=UjK3SWej_4E>.  YouTube. Jamaican authorities discuss illegal pork imports [Internet]. 2024 [cited Nov-Dec 2024]. Available from: <https://www.youtube.com/watch?v=-p4LtMu_-FM>.  Facebook. Another group of Haitian migrants reportedly arrived in Jamaica [Internet]. 2024 [cited Nov-Dec 2024]. Available from: <https://www.facebook.com/IrieFmJA/videos/another-group-of-haitian-migrants-reportedly-arrived-in-jamaica-on-saturday-afte/565612815815355/>.  Jamaica Gleaner. Illegal pork imports suspected; Agriculture Ministry moves to protect industry [Internet]. 2024 Sep 11 [cited Nov-Dec 2024]. Available from: <https://jamaica-gleaner.com/article/business/20240911/illegal-pork-imports-suspected-agriculture-ministry-moves-protect-industry#google_vignette>.  IOM. Jamaica: Migration and border control [Internet]. 2024 [cited Nov-Dec 2024]. Available from: <https://www.iom.int/countries/jamaica>.  Radio Jamaica News. More than 80 Haitians who arrived in Jamaica since last year repatriated [Internet]. 2024 Sep 8 [cited Nov-Dec 2024]. Available from: <https://radiojamaicanewsonline.com/local/more-than-80-haitians-who-arrived-in-jamaica-since-last-year-repatriated>.  Embassy of Jamaica. Customs regulations for visitors [Internet]. 2024 [cited Nov-Dec 2024]. Available from: <https://www.embassyofjamaica.org/visitors/customs_regulations.htm>.  TripAdvisor. Food and drinks brought into Jamaica [Internet]. 2024 [cited Nov-Dec 2024]. Available from: <https://www.tripadvisor.com/ShowTopic-g147311-i93-k162959-Food_and_drinks_brought_into_Jamaica-Montego_Bay_Saint_James_Parish_Jamaica.html>.  Ministry of Agriculture, Jamaica. Domestic import of animal and animal products [Internet]. 2023 Jun 23 [cited Nov-Dec 2024]. Available from: <https://www.moa.gov.jm/sites/default/files/UPDATED-JUNE-23-2023-DOMESTIC-IMPORT-OF-ANIMAL-and-animal-products-document-for-NMIA-and-MBJ.pdf>. |
| Martinique | Unknown, Probable | L | Culturally, Martinique is similar to Haiti potentially leading to the movement of Haitians to this island. Haitian migrants are smuggled to Martinique through the transit territory of Guadeloupe. Martinique is part of the European Union, which prohibits the importation of meat products unless in specific amounts from certain countries. Limited documentation on biosecurity measures specific to migrant-related risks exists, despite heightened border surveillance and collaboration with neighboring territories like Guadeloupe. | | | Dominica News Online. Migrant smuggling from Dominica a major headache for the French government [Internet]. 2024 [cited Nov-Dec 2024]. Available from: <https://dominicanewsonline.com/news/governance/migrant-smuggling-from-dominica-a-major-headache-for-the-french-government/>.  NationMaster. Martinique transport profile [Internet]. 2024 [cited Nov-Dec 2024]. Available from: <https://www.nationmaster.com/country-info/profiles/Martinique/Transport>.  Travel Weekly. Martinique: Caribbean with a French flavor [Internet]. 2024 [cited Nov-Dec 2024]. Available from: <https://www.travelweekly.com/Cruise/Martinique-Caribbean-with-a-French-flavor/302488>.  VisaHQ. Martinique customs regulations [Internet]. 2024 [cited Nov-Dec 2024]. Available from: <https://www.visahq.com/martinique/customs/#:~:text=Non%2Dcommercial%20item%20are%20of,%E2%80%A2>.  Reddit. Thoughts of Martiniquans [Internet]. 2024 [cited Nov-Dec 2024]. Available from: <https://www.reddit.com/r/haiti/comments/z14hf4/thoughts_of_martiniquans/?rdt=59814>.  Ed Hamilton. Import restrictions for Martinique [Internet]. 2024 [cited Nov-Dec 2024]. Available from: <https://www.ed-hamilton.com/resources/import-restrictions/>. |
| Mexico | Probable | M | Mexico has established tracking systems at formal entry points, however, illegal imports through migration and trade make monitoring a challenge.  Recent measures, such as strengthened inspections of aircraft and ships from the Dominican Republic, include incineration of leftovers and disinfection mats on international flights. While biosecurity at ports is robust, small amounts of meat from non-affected areas are still confiscated, indicating informal importation. Most Haitian migrants at Mexico's borders emigrated years ago, particularly following the 2009 earthquake, with many residing in South America and Central America before traveling north. There has been a recent influx of recent Haitian immigrants traveling from South America, specifically from Nicaragua, to the Mexican-US border, with many choosing to remain in Mexico. Meanwhile, Mexico has seen an increase in Chinese immigration, doubling the issuance of temporary residency visas to 5,070 in 2023. | | | Migration Policy Institute. Haitian migration through the Americas [Internet]. 2024 [cited Nov-Dec 2024]. Available from: <https://www.migrationpolicy.org/article/haitian-migration-through-americas>.  ArcGIS StoryMaps. Haitian migration routes [Internet]. 2024 [cited Nov-Dec 2024]. Available from: <https://storymaps.arcgis.com/stories/c308e7961ae149e78cb8e2b3c31b3b53>.  FHD Global. The Haitian diaspora [Internet]. 2024 [cited Nov-Dec 2024]. Available from: <https://fhd.global/the-haitian-diaspora/>.  BBC News. Haiti migrants: Thousands gather under bridge at US-Mexico border [Internet]. 2021 Sep 24 [cited Nov-Dec 2024]. Available from: <https://www.bbc.com/news/world-latin-america-58673578>.  ResearchGate. Route of Haitian immigrants [Internet]. 2024 [cited Nov-Dec 2024]. Available from: <https://www.researchgate.net/figure/Route-of-Haitian-immigrants-to_fig5_332798715>.  Associated Press. Migrants protest for asylum in Mexico [Internet]. 2023 Sep 22 [cited Nov-Dec 2024]. Available from: <https://apnews.com/article/mexico-migrants-cuban-haitian-honduran-protest-asylum-c70b0812f0f3a647d9af9729da6ff0d3>.  Reuters. Mexico feels strain as Haitian refugees caught in limbo mark time [Internet]. 2023 Apr 17 [cited Nov-Dec 2024]. Available from: <https://www.reuters.com/world/americas/mexico-feels-strain-haitian-refugees-caught-limbo-mark-time-2023-04-17/>.  Pig333. Mexico reinforces controls to prevent the entry of ASF [Internet]. 2024 [cited Nov-Dec 2024]. Available from: <https://www.pig333.com/latest_swine_news/mexico-reinforces-controls-to-prevent-the-entry-of-asf_18957/>.  Gobierno de México. Mexico activates the Integral Biosecurity Plan facing the ASF threat [Internet]. 2024 [cited Nov-Dec 2024]. Available from: <https://www.gob.mx/cms/uploads/attachment/file/659710/Mexico_activates_the_Integral_Biosecurity_plan__facing_the_African_Swine_Fever_ASF_threat.pdf>.  Business Standard. Chinese migrants flock to Mexico in search of jobs and freedom [Internet]. 2024 Sep 5 [cited Nov-Dec 2024]. Available from: <https://www.business-standard.com/world-news/chinese-migrants-flock-to-mexico-in-search-of-jobs-and-taste-of-freedom-124090500061_1.html>.  WOLA. Migration country by country at the U.S.-Mexico border [Internet]. 2022 Nov [cited Nov-Dec 2024]. Available from: <https://www.wola.org/2022/11/migration-country-by-country-at-the-u-s-mexico-border/>.  Migration Data Portal. Key migration figures in the Americas [Internet]. 2024 [cited Nov-Dec 2024]. Available from: <https://www.migrationdataportal.org/americas/key-figures>.  National Hog Farmer. Mexico and U.S. join efforts to eradicate ASF in Caribbean countries [Internet]. 2024 [cited Nov-Dec 2024]. Available from: <https://www.nationalhogfarmer.com/biosecurity/mexico-u-s-join-efforts-to-eradicate-asf-in-caribbean-countries>.  Pig333. Mexico actions and measures to prevent the entry of ASF [Internet]. 2024 [cited Nov-Dec 2024]. Available from: <https://www.pig333.com/latest_swine_news/mexico-actions-and-measures-to-prevent-the-entry-of-asf_14917/>.  National Hog Farmer. Canada, Mexico, and U.S. unite to keep North America ASF-free [Internet]. 2024 [cited Nov-Dec 2024]. Available from: <https://www.nationalhogfarmer.com/hog-health/canada-mexico-and-u-s-unite-to-keep-north-america-asf-free>.  PLOS One. A study on migration patterns in the Americas [Internet]. 2024 [cited Nov-Dec 2024]. Available from: <https://journals.plos.org/plosone/article?id=10.1371/journal.pone.0029505>.  Voice of America. Giving up on U.S.: Haitian migrants opt for the Mexican dream [Internet]. 2024 [cited Nov-Dec 2024]. Available from: <https://www.voanews.com/a/giving-up-on-us-haitian-migrants-opt-for-mexican-dream-/7502437.html>. |
| Montserrat | Unknown, Unlikely | L | While reports indicate some instances of meat being brought into the island, there are no specific prohibitions on pork products. Recent human smuggling incidents, including vessels carrying Haitian nationals, highlight potential vulnerabilities in border security, which could indirectly increase ASF risk through contaminated food products. There is limited surveillance data and unclear enforcement of biosecurity measures. | | | Government of Montserrat. 18 Haitian Nationals Repatriated from Montserrat [Internet]. 2024 Feb 26 [cited Nov-Dec 2024]. Available from: <https://www.gov.ms/2024/02/26/18-haitian-nationals-repatriated-from-montserrat/>  ECCB. Selected Tourism Statistics [Internet]. [cited Nov-Dec 2024]. Available from: <https://www.eccb-centralbank.org/statistics-category/external-sector/selected-tourism-statistics>  TripAdvisor. Bringing food/meat - Montserrat Forum [Internet]. 2015 Sep 30 [cited Nov-Dec 2024]. Available from: <https://www.tripadvisor.com/ShowTopic-g147333-i1913-k8904197-Bringing_food_meat-Montserrat.html>  VisaHQ. Montserrat Customs Regulations [Internet]. [cited Nov-Dec 2024]. Available from: <https://www.visahq.com/montserrat/customs/> |
| Nicaragua | Unknown, Probable | L | Nicaragua has a surveillance measure but this is offset by vulnerabilities in migration, human smuggling, and the economic state of the country. The country enforces biosecurity through quarantine drills and legislation, yet an influx of migrants from Cuba on charter flights and relaxed visa policies along with the political situation has cause for concern. After removing visa restrictions for Cubans in 2022, Nicaragua experienced an increase in migration from Cuba and Haiti. Migrants take chartered flights, where tickets are purchased through unofficial channels, from Cuba to Nicaragua with some stopping in Port-au-Prince. There is also concern with the current economic recession and political unrest resulting in mass emigration of Nicaraguans.  According to the US Department of State’s 2024 Trafficking Report, Nicaragua has not met the minimum standards for eliminating human trafficking, and has made little effort to do so, revealing potential gaps in biosecurity where smuggled humans may carry diseases across borders.  Nicaragua did conduct training to strengthen biosecurity and surveillance measures in 2019.  Small volumes of informal imports have been seized, and fresh food is prohibited in luggage and mail. | | | Ministerio de Economía Familiar, Comunitaria, Cooperativa y Asociativa. Gobierno de Nicaragua fortalece sistema de vigilancia en sanidad animal [Internet]. [cited Nov-Dec 2024]. Available from: <https://www.economiafamiliar.gob.ni/websitemefcca-mvc/noticia-gobierno-nicaragua-fortalece-sistema-vigilancia-sanidad-animal/521>  Asamblea Nacional de Nicaragua. Legislación de Nicaragua [Internet]. [cited Nov-Dec 2024]. Available from: <http://legislacion.asamblea.gob.ni/Normaweb.nsf/3133c0d121ea3897062568a1005e0f89/6d43fff3e305b79d062584a500520b26?OpenDocument>  Associated Press. Cuba-Nicaragua migration charter flights [Internet]. 2023 Dec 15 [cited Nov-Dec 2024]. Available from: <https://apnews.com/article/cuba-nicaragua-migration-charter-flights-daniel-ortega-3abf2fc16e51e86eb8b25c913b8ec464>  Migration Policy Institute. Record emigration from Nicaragua amid crisis [Internet]. [cited Nov-Dec 2024]. Available from: <https://www.migrationpolicy.org/article/record-emigration-nicaragua-crisis>  Migration Policy Institute. Haitian migration through the Americas [Internet]. [cited Nov-Dec 2024]. Available from: <https://www.migrationpolicy.org/article/haitian-migration-through-americas>  National Public Radio. How Nicaragua is weaponizing immigration to the U.S. [Internet]. 2024 Jan 4 [cited Nov-Dec 2024]. Available from: <https://www.npr.org/2024/01/04/1222951949/how-nicaragua-is-weaponizing-immigration-to-the-u-s>  Migrants & Refugees Section. Nicaragua Country Profile [Internet]. [cited Nov-Dec 2024]. Available from: <https://migrants-refugees.va/it/wp-content/uploads/sites/3/2022/03/2022-CP-Nicaragua.pdf>  Tico Times. Surge in Cuban, Haitian U.S. migration via Nicaragua [Internet]. 2023 Nov 23 [cited Nov-Dec 2024]. Available from: <https://ticotimes.net/2023/11/23/surge-in-cuban-haitian-us-migration-via-nicaragua>  Tico Times. Cubans rally in front of Costa Rican consulate for transit visas [Internet]. 2022 Feb 22 [cited Nov-Dec 2024]. Available from: <https://ticotimes.net/2022/02/22/cubans-rally-in-front-of-costa-rican-consulate-for-transit-visas>  Confidencial. Inside the operation of smuggling migrants to the U.S. via Nicaragua’s airport [Internet]. 2024 Jan 10 [cited Nov-Dec 2024]. Available from: <https://confidencial.digital/english/inside-the-operation-of-smuggling-migrants-to-the-u-s-via-nicaraguas-airport/>  Havana Times. The large-scale flow of Cubans to Nicaragua continues [Internet]. 2024 Feb 5 [cited Nov-Dec 2024]. Available from: <https://havanatimes.org/cuba/the-large-scale-flow-of-cubans-to-nicaragua-continues/> |
| Panama | Probable | M | Panama has a Likely risk of ASF introduction despite preventative measures, including a declared zoosanitary alert, import prohibitions on pork from ASF-affected countries, and strengthened biosecurity at ports and airports. Due to the vast amount of migrants passing through Panama there are reported difficulties in managing unregulated movements of people and biosecurity gaps.  As a key transit point for migrants, including those from ASF-affected regions, Panama’s location presents logistical challenges. The Darién Gap complicates enforcement, with smuggling and informal importation difficult to control. In 2021, 91,300 migrants,mostly Haitians, crossed the Darien Gap jungle to enter Panama. Surveillance has improved, but has demonstrated that there is meat from the DR being illegally brought into the country. Small volumes of informal imports pass through, with origins often unrecorded. Animal products must have phytosanitary certification for customs clearance, but gaps in enforcement persist. | | | Organismo Internacional Regional de Sanidad Agropecuaria (OIRSA). African Swine Fever Report – Revised Edition [Internet]. 2020 Jul 1 [cited Nov-Dec 2024]. Available from: <https://www.oirsa.org/contenido/2020/AR_PPA_Edici%C3%B3n%20revisada%2001_07_20.pdf>  Migration Policy Institute. Haitian migration through the Americas [Internet]. [cited Nov-Dec 2024]. Available from: <https://www.migrationpolicy.org/article/haitian-migration-through-americas>  Food and Agriculture Organization (FAO). Panama: Animal health legislation [Internet]. [cited Nov-Dec 2024]. Available from: <https://faolex.fao.org/docs/pdf/pan190542.pdf>  3tres3. Panamá podría restringir la importación de carne de cerdo [Internet]. 2019 Aug 20 [cited Nov-Dec 2024]. Available from: <https://www.3tres3.com/ultima-hora/panama-podria-restringir-la-importacion-de-carne-de-cerdo_41122/>  El Periódico. Panamá prohíbe importar cerdos y derivados de países con peste porcina [Internet]. 2019 Aug 21 [cited Nov-Dec 2024]. Available from: <https://www.elperiodico.com/es/economia/20190821/panama-prohibe-importar-cerdos-derivados-paises-peste-porcina-7600545>  Refugees International. After the Darién: Aid and pathways for migrants in Panama and Costa Rica [Internet]. [cited Nov-Dec 2024]. Available from: <https://www.refugeesinternational.org/reports-briefs/after-the-darien-aid-and-pathways-for-migrants-in-panama-and-costa-rica/>  Panamá América. Detectan en Bocas del Toro 80 libras de carne de cerdo proveniente de República Dominicana [Internet]. 2023 Sep 15 [cited Nov-Dec 2024]. Available from: <https://www.panamaamerica.com.pa/provincias/detectan-en-bocas-del-toro-80-libras-de-carne-de-cerdo-proveniente-de-republica>  International Organization for Migration (IOM). More than 91,000 migrants have crossed the Darién Gap on their way to North America this year [Internet]. 2024 Apr 3 [cited Nov-Dec 2024]. Available from: <https://www.iom.int/news/more-91000-migrants-have-crossed-darien-gap-way-north-america-year>  Council on Foreign Relations (CFR). Crossing the Darién Gap: Migrants risk death on journey to U.S. [Internet]. 2023 Oct 12 [cited Nov-Dec 2024]. Available from: <https://www.cfr.org/article/crossing-darien-gap-migrants-risk-death-journey-us>  Tocumen International Airport. Customs and immigration regulations [Internet]. [cited Nov-Dec 2024]. Available from: <https://www.tocumenpanama.aero/index.php/inmigracion/aduana>  TripAdvisor. Bringing food into Panama – Tocumen airport [Internet]. 2019 Sep 5 [cited Nov-Dec 2024]. Available from: <https://www.tripadvisor.com/ShowTopic-g294480-i1194-k12879096-Bringing_food_into_Panama_Tocumen_airport-Panama_City_Panama_Province.html>  On The Go Tours. Travel tips and useful information for Panama [Internet]. [cited Nov-Dec 2024]. Available from: <https://www.onthegotours.com/Panama/Guides/Travel-Tips-and-Useful-Info#:~:text=You%20may%20bring%20in%20gifts,explosives%2C%20narcotics%20and%20hazardous%20materials>. |
| Puerto Rico | Probable | M | Puerto Rico has a Likely risk of ASF introduction due to informal pork product imports and migrant smuggling through maritime routes, particularly from the Dominican Republic and Haiti.  Due to the proximity of Puerto Rico to Haiti and the DR, and it being a US Territory, it has become an important island for smugglers and migrants. In 2021, over 65,000 Puerto Ricans traveled to the DR, and 16,000 Dominicans traveled to Puerto Rico.  Since the DR is only 80 miles from Puerto Rico, there is a large Dominican population living in Puerto Rico, who may import cultural items. Along with this legal entrance of visitors, there are numerous reports of Haitian immigrants illegally entering Puerto Rico. There is a robust surveillance program in place due to the establishment of a Protection Zone in 2021. This allows for USDA-APHIS to work closely with partners in Puerto Rico to increase education, outreach, and biosecurity measures. However, there are also reports of informal pork products being confiscated from ferryboats. | | | Virgin Islands Department of Agriculture. The Virgin Islands Department of Agriculture continues disease surveillance of all swine and chicken avian farms in the territory [Internet]. [cited Nov-Dec 2024]. Available from: <https://doa.vi.gov/the-virgin-islands-department-of-agriculture-continues-disease-surveillance-of-all-swine-and-chicken-avian-farms-in-the-territory/>  Euro Meat News. Federal order issued to protect Puerto Rico from ASF [Internet]. 2021 Oct 5 [cited Nov-Dec 2024]. Available from: <https://www.euromeatnews.com/Article-Federal-order-issued-to-protect-Puerto-Rico-from-ASF/4962>  Virgin Islands Department of Agriculture. African Swine Fever (ASF) [Internet]. [cited Nov-Dec 2024]. Available from: <https://doa.vi.gov/asf/>  World Organisation for Animal Health (WOAH). ASF protection zone in the U.S. Caribbean [Internet]. 2021 Oct [cited Nov-Dec 2024]. Available from: <https://www.woah.org/app/uploads/2021/10/2021-10-usa-asf-pz-uscaribbean.pdf>  United States Department of Agriculture (USDA). African Swine Fever Part 2: Safeguarding the future [Internet]. 2022 Oct 14 [cited Nov-Dec 2024]. Available from: <https://www.usda.gov/media/blog/2022/10/14/african-swine-fever-part-2-safeguarding-future>  USDA Animal and Plant Health Inspection Service (APHIS). USDA takes additional steps to prevent African Swine Fever in Puerto Rico and U.S. Virgin Islands [Internet]. 2023 Oct 6 [cited Nov-Dec 2024]. Available from: <https://content.govdelivery.com/accounts/USDAAPHIS/bulletins/3741a70>  United States Coast Guard. Coast Guard rescues 11 Haitian migrants left stranded by smugglers on Monito Island [Internet]. 2024 Jan 10 [cited Nov-Dec 2024]. Available from: <https://www.news.uscg.mil/Press-Releases/Article/3903920/coast-guard-rescues-11-haitian-migrants-left-stranded-by-smugglers-on-monito-is/>  Federal Reserve Bank of New York. Population lost: Puerto Rico’s troubling out-migration [Internet]. 2015 Apr 13 [cited Nov-Dec 2024]. Available from: <https://libertystreeteconomics.newyorkfed.org/2015/04/population-lost-puerto-ricos-troubling-out-migration/>  U.S. Customs and Border Protection (CBP). Puerto Rico and the U.S. Virgin Islands travel information [Internet]. [cited Nov-Dec 2024]. Available from: <https://www.cbp.gov/node/382902>  Virgin Islands Daily News. As DR ramps up Haitian deportations, smugglers dump over 100 migrants in Puerto Rico [Internet]. 2024 Feb 18 [cited Nov-Dec 2024]. Available from: <https://www.virginislandsdailynews.com/ap/as-dr-ramps-up-haitian-deportations-smugglers-dump-over-100-migrants-in-puerto-rico/article_97366cbc-81e1-11ef-b2df-a76126bed98b.html>  United States Coast Guard. Coast Guard returns 24 migrants to the Dominican Republic following vessel interdiction [Internet]. 2023 May 20 [cited Nov-Dec 2024]. Available from: <https://www.news.uscg.mil/Press-Releases/Article/3413380/coast-guard-returns-24-migrants-to-the-dominican-republic-following-vessel-inte/> |
| Saba | Unknown, Unlikely | L | Results are due to the location of Saba along with the very limited information gathered. | | | Rijksdienst Caribisch Nederland. Stricter supervision of illegal residence on Bonaire, Saba, and St. Eustatius [Internet]. 2023 Jul 6 [cited Nov-Dec 2024]. Available from: <https://english.rijksdienstcn.com/latest/news/2023/july/06/stricter-supervision-of-illegal-residence-on-bonaire-saba-and-st.-eustatius> |
| Saint Barthelemy | Unknown, Unlikely | L | No pigs or miniscule number present in the territory. | | | European Commission. Agri-food trade: St. Barthélemy [Internet]. 2023 May [cited Nov-Dec 2024]. Available from: <https://agriculture.ec.europa.eu/system/files/2023-05/agrifood-st-barthelemy_en.pdf>  Frommer's. Entry requirements & customs: St. Barts [Internet]. [cited Nov-Dec 2024]. Available from: <https://www.frommers.com/destinations/st-barts/planning-a-trip/entry-requirements--customs>  Ed Hamilton & Co. Import restrictions [Internet]. [cited Nov-Dec 2024]. Available from: <https://www.ed-hamilton.com/resources/import-restrictions/> |
| Saint Lucia | Unknown, Unlikely | L | Meat imports are subject to inspection, but limited quantities of USDA-approved frozen meats are permitted. Reports suggest that raw meat is inspected but may still be allowed, and informal imports from French territories contribute to potential exposure. Human trafficking and migration from Haiti and other regions add to biosecurity concerns. Limited available data on active surveillance and enforcement measures. | | | United States Department of State. Trafficking in persons report: Saint Lucia [Internet]. 2014 [cited Nov-Dec 2024]. Available from: <https://2009-2017.state.gov/j/tip/rls/tiprpt/countries/2014/226820.htm>  Eastern Caribbean Central Bank. Selected tourism statistics [Internet]. [cited Nov-Dec 2024]. Available from: <https://www.eccb-centralbank.org/statistics-category/external-sector/selected-tourism-statistics>  International Organization for Migration. Strengthening migration regulatory and legal frameworks in Saint Lucia and promoting good migration governance [Internet]. [cited Nov-Dec 2024]. Available from: <https://www.iom.int/project/strengthening-migration-regulatory-and-legal-frameworks-st-lucia-and-promoting-good-migration-governance>  Saint Lucia Customs and Excise Department. Traveller’s information [Internet]. [cited Nov-Dec 2024]. Available from: <https://www.customs.gov.lc/travellers-information.php>  Government of Saint Lucia. Customs regulations on food imports [Internet]. [cited Nov-Dec 2024]. Available from: <https://archive.stlucia.gov.lc/faq/customs_regualtions_on_food_imports.htm#:~:text=There%20should%20be%20no%20difficulties,at%20the%20time%20you%20arrive>.  TripAdvisor. Bringing food to Saint Lucia? [Internet]. [cited Nov-Dec 2024]. Available from: <https://www.tripadvisor.com/ShowTopic-g147342-i247-k11439708-Bringing_food-St_Lucia.html>  TripAdvisor. Packing meat in a suitcase for Saint Lucia? [Internet]. [cited Nov-Dec 2024]. Available from: <https://www.tripadvisor.com/ShowTopic-g147342-i247-k14624316-Packing_Meat_in_Suitcase-St_Lucia.html>  Ed Hamilton & Co. Import restrictions [Internet]. [cited Nov-Dec 2024]. Available from: <https://www.ed-hamilton.com/resources/import-restrictions/> |
| Saint Martin | Unknown, Probable | L | Saint Martin the French Territory has enacted a continual ban of pork and pork products from ASF infected countries and documented surveillance efforts at ports of entry (80).  Sint Maarten, the Dutch Territory, is on the same island as Saint Martin. The border shared between these two territories is unregulated allowing for free movement between them (81). Illegal migrants are flown into Sint Maarten before being smuggled to USVI on ships (38). Due to the porous border migrants could freely pass from Sint Maarten into Saint Martin. Surveillance measures and pork product bans mitigate some of the risk of ASF introduction, but porous borders and illegal migrants make Saint Martin a Likely risk. | | | SMN News. Continued ban on all pork products originating from the Dominican Republic due to positive African swine fever contaminated products [Internet]. 2024 [cited Nov-Dec 2024]. Available from: <https://smn-news.com/index.php/st-maarten-st-martin-news/39650-continued-ban-on-all-pork-products-originating-from-the-dominican-republic-due-to-positive-african-swine-fever-contaminated-products.html>  VisaHQ. Saint Martin customs regulations [Internet]. [cited Nov-Dec 2024]. Available from: <https://www.visahq.com/saint-martin/customs/#!export-regulations>  International Organization for Migration. Sint Maarten needs assessment: Migration governance [Internet]. [cited Nov-Dec 2024]. Available from: <https://programamesoamerica.iom.int/sites/default/files/sint-maarten-needs-assessment-migration-governance.pdf>  International Organization for Migration. Environmental migration and disaster displacement: Case studies and policy insights [Internet]. [cited Nov-Dec 2024]. Available from: <https://environmentalmigration.iom.int/sites/g/files/tmzbdl1411/files/documents/2023-09/469cbfaf0.pdf>  TripAdvisor. Bringing food into Sint Maarten? [Internet]. [cited Nov-Dec 2024]. Available from: <https://www.tripadvisor.com/ShowTopic-g1606589-i15357-k10369640-o10-Bringing_Food_into_SXM-Saint_Martin_St_Martin_St_Maarten.html>  TripAdvisor. Can you bring food and alcohol in checked bags to Sint Maarten? [Internet]. [cited Nov-Dec 2024]. Available from: <https://www.tripadvisor.com/ShowTopic-g147346-i222-k13537772-Can_You_Bring_Food_Alcohol_in_Checked_Bags-St_Martin_St_Maarten.html>  Financial Investigation Agency British Virgin Islands. Migrant smuggling: Extent and impact in the Virgin Islands [Internet]. [cited Nov-Dec 2024]. Available from: <https://fiabvi.vg/Portals/0/DNNGalleryPro/uploads/2024/10/3/MigrantSmuggling-ExtentandImpactintheVIforPublicUse(FINAL).pdf>  Ed Hamilton & Co. Import restrictions [Internet]. [cited Nov-Dec 2024]. Available from: <https://www.ed-hamilton.com/resources/import-restrictions/> |
| Sint Eustatius | Unknown, Unlikely | L | Limited data on active surveillance, enforcement measures, and minimal information on informal imports. | | | Rijksdienst Caribisch Nederland. Stricter supervision of illegal residence on Bonaire, Saba, and St. Eustatius [Internet]. 2023 Jul 6 [cited Nov-Dec 2024]. Available from: <https://english.rijksdienstcn.com/latest/news/2023/july/06/stricter-supervision-of-illegal-residence-on-bonaire-saba-and-st.-eustatius> |
| Sint Maarten | Unknown, Probable | L | *See Saint Martin above* | | | Government of Sint Maarten. Temporary ban on all pork products originating from the Dominican Republic due to African swine fever contamination [Internet]. 2024 Oct 3 [cited Nov-Dec 2024]. Available from: <https://www.facebook.com/SXMGOV/posts/-temporary-ban-on-all-pork-products-originating-from-the-dominican-republic-due-/2007793802725268/>  Financial Investigation Agency of the British Virgin Islands. Migrant smuggling: Extent and impact in the Virgin Islands [Internet]. 2024 Oct 3 [cited Nov-Dec 2024]. Available from: <https://fiabvi.vg/Portals/0/DNNGalleryPro/uploads/2024/10/3/MigrantSmuggling-ExtentandImpactintheVIforPublicUse(FINAL).pdf>  Ed Hamilton & Co. Import restrictions [Internet]. [cited Nov-Dec 2024]. Available from: <https://www.ed-hamilton.com/resources/import-restrictions/>  Government of Canada. Travelling to the Schengen area [Internet]. [cited Nov-Dec 2024]. Available from: <https://travel.gc.ca/travelling/schengen-area> |
| St Kitts and Nevis | Unlikely | M | St. Kitts and Nevis has strict import regulations, including veterinary import permits and inspection protocols. They enforce a ban on meat imports from ASF-affected regions while allowing limited personal meat imports under 50 lbs from countries free of FMD and HPAI. Strong biosecurity measures, regional collaboration, and public education efforts further reduce the risk. There are some reports of migrant smuggling. | | | St. Kitts and Nevis Information Service. St. Kitts and Nevis Parliament sends strong message to Caribbean human smuggling gangs [Internet]. 2024 Jun 19 [cited Nov-Dec 2024]. Available from: <https://www.sknis.gov.kn/2024/06/19/st-kitts-and-nevis-parliament-sends-strong-message-to-caribbean-human-smuggling-gangs/>  St. Kitts and Nevis Information Service. African Swine Flu Bulletin [Internet]. 2023 Dec 20 [cited Nov-Dec 2024]. Available from: <https://www.sknis.gov.kn/2023/12/20/african-swine-flu-bulletin/>  St. Kitts and Nevis Customs and Excise Department. Veterinary import permit for swine [Internet]. [cited Nov-Dec 2024]. Available from: <https://skncustoms.com/Forms%20and%20Guides/VETERINARY%20IMPORT%20PERMIT%20FOR%20SWINE.pdf>  Eastern Caribbean Central Bank. Selected tourism statistics [Internet]. [cited Nov-Dec 2024]. Available from: <https://www.eccb-centralbank.org/statistics-category/external-sector/selected-tourism-statistics>  St. Kitts and Nevis Customs and Excise Department. Prohibited and restricted goods [Internet]. [cited Nov-Dec 2024]. Available from: <https://skncustoms.com/pdfs/PROHIBITED%20AND%20RESTRICTED%20GOODS.pdf>  Government of St. Kitts and Nevis. Agriculture permits [Internet]. [cited Nov-Dec 2024]. Available from: <https://www.gov.kn/agriculture-permits/#:~:text=Each%20species%20has%20its%20own,permit%20would%20not%20be%20required>.  TripAdvisor. Anything you can't bring into St. Kitts? [Internet]. [cited Nov-Dec 2024]. Available from: <https://www.tripadvisor.com/ShowTopic-g147374-i541-k8287042-Anything_you_can_t_bring_into_St_kitts-St_Kitts_St_Kitts_and_Nevis.html>  Ed Hamilton & Co. Import restrictions [Internet]. [cited Nov-Dec 2024]. Available from: <https://www.ed-hamilton.com/resources/import-restrictions/> |
| St. Vincent and the Grenadines | Unknown, Unlikely | L | St. Vincent and the Grenadines remains on "Red Alert" for ASF, enforcing strict biosecurity measures such as 100% quarantine checks and import bans from ASF-affected regions. There are gaps in tracking informal imports and the effectiveness of enforcement. Personal meat imports require an import permit and must meet specific conditions. There are reports of ongoing smuggling activities and illegal immigration from neighboring islands. | | | Government of Saint Vincent and the Grenadines. Official website [Internet]. [cited Nov-Dec 2024]. Available from: <https://www.gov.vc/>  Eastern Caribbean Central Bank. Selected tourism statistics [Internet]. [cited Nov-Dec 2024]. Available from: <https://www.eccb-centralbank.org/statistics-category/external-sector/selected-tourism-statistics>  Discover Saint Vincent and the Grenadines. Official tourism website [Internet]. [cited Nov-Dec 2024]. Available from: <https://www.discoversvg.com/>  TripAdvisor. Bringing food into Saint Vincent? [Internet]. [cited Nov-Dec 2024]. Available from: <https://www.tripadvisor.com/ShowTopic-g147379-i1174-k14521845-Bringing_food_into_St_Vincent-Saint_Vincent_and_the_Grenadines.html>  VisaHQ. Saint Vincent and the Grenadines customs regulations [Internet]. [cited Nov-Dec 2024]. Available from: <https://www.visahq.com/saint-vincent-and-the-grenadines/customs/>  Ed Hamilton & Co. Import restrictions [Internet]. [cited Nov-Dec 2024]. Available from: <https://www.ed-hamilton.com/resources/import-restrictions/> |
| Suriname | Unknown, Probable | L | Suriname has an influx of migrants and asylum-seekers, many of whom face exploitation in informal sectors and lack legal protection. Corruption and complicity among some officials further complicate border control and anti-trafficking measures. With limited surveillance and incomplete data, the exact risk level remains uncertain. The vast, under-patrolled borders, combined with being understaffed and overwhelmed security forces, make enforcement of biosecurity measures difficult. Although there is currently low migration and no reported informal pork product imports, smuggling remains a concern. | | | 94. Diálogo. (2024). *Suriname: Strengthening border protection from illegal activities*.<https://dialogo-americas.com/articles/suriname-strengthening-border-protection-from-illegal-activities/>  95. U.S. Department of State. (2024). *2024 trafficking in persons report: Suriname*.<https://www.state.gov/reports/2024-trafficking-in-persons-report/suriname/#:~:text=Traffickers%20target%20the%20increasing%20influx> |
| Trinidad and Tobago | Unknown, Probable | L | Trinidad and Tobago are a regional trade and travel hub, which may lead to smuggling activities. Strict regulations require a valid permit from the Chief Technical Officer of Agriculture for meat imports, and uncooked meat is not permitted through customs. However, there are reports of informal importation in forums, but they are outdated. | | | TripAdvisor. Bringing food into Tobago discussion [Internet]. [cited Nov-Dec 2024]. Available from: <https://www.tripadvisor.com/ShowTopic-g147387-i548-k247341-Bringing_food_into_Tobago-Trinidad_and_Tobago.html>  International Organization for Migration. Trinidad and Tobago country profile [Internet]. [cited Nov-Dec 2024]. Available from: <https://www.iom.int/countries/trinidad-and-tobago>  Government of Trinidad and Tobago. Immigration Act Chapter 18:01 [Internet]. [cited Nov-Dec 2024]. Available from: <https://agla.gov.tt/downloads/laws/67.02.pdf> |
| Turks and Caicos | Probable | M | Turks and Caicos has a Likely risk of ASF introduction due to its proximity to ASF-affected regions and significant human smuggling activities. Regional collaboration with organizations such as USDA and IICA has bolstered biosecurity efforts, yet the increasing number of intercepted illegal vessels poses ongoing challenges, straining the resources of the government of Turks and Caicos.  Reports indicate informal imports of live pigs and pork products from Haiti and the Dominican Republic. While commercial quantities of meat require permits, most grocery-purchased food is legally permitted in personal luggage without the need for a permit. There are reports of weekly influxes of boatloads of Haitians fleeing to Turks and Caicos and being detained. As of August 2023, 3030 immigrants were detained. The movement of these migrants through smuggling or other means is putting a strain on their immigration resources and border patrols. | | | Royal Turks and Caicos Islands Police Force. Two hundred and four illegal migrants intercepted at sea [Internet]. Providenciales (TCA): RTCIPF; 2024. [cited 2024 Nov-Dec]. Available from: <https://www.tcipolice.tc/two-hundred-and-four-illegal-migrants-intercepted-at-sea/>  TC Weekly News. TCI cracks down on illegal immigration: repeat offenders sentenced [Internet]. Grand Turk (TCA): TC Weekly News; 2024 Feb. [cited 2024 Nov-Dec]. Available from: <https://tcweeklynews.com/tci-cracks-down-on-illegal-immigration-repeat-offenders-sentenced-p14574-155.htms>  Sun TCI. Turks and Caicos Islands delegation attends Caribbean Week of Agriculture [Internet]. Providenciales (TCA): Sun TCI; 2024 Feb. [cited 2024 Nov-Dec]. Available from: <https://suntci.com/turks-and-caicos-islands-delegation-attends-caribbean-week-of-agriculture-p9889-129.htm>  Visit TCI. Importing animal and plant products [Internet]. Providenciales (TCA): Visit Turks and Caicos Islands; [date unknown] [cited 2024 Nov-Dec]. Available from: <https://www.visittci.com/travel-info/entry-requirements/importing-animal-and-plant-products>  Migration Policy Institute. Haitian migration through the Americas: a decade in review [Internet]. Washington (DC): MPI; [date unknown] [cited 2024 Nov-Dec]. Available from: <https://www.migrationpolicy.org/article/haitian-migration-through-americas>  The Guardian. Hundreds of Haitian migrants flee to Turks and Caicos amid crisis at home [Internet]. London (GBR): The Guardian; 2023 Jan 5. [cited 2024 Nov-Dec]. Available from: <https://www.theguardian.com/world/2023/jan/05/turks-and-caicos-haitian-migrants-immigration>  TC Weekly News. Migration of illegal Haitians to TCI hits new high as conditions worsen in Haiti [Internet]. Grand Turk (TCA): TC Weekly News; 2023. [cited 2024 Nov-Dec]. Available from: <https://tcweeklynews.com/migration-of-illegal-haitians-to-tci-hits-for-as-conditions-in-h-p13448-127.htm>  Loop News Cayman. Turks and Caicos intercepts sixth vessel of Haitians in 2023 [Internet]. George Town (CYM): Loop News Cayman; 2023. [cited 2024 Nov-Dec]. Available from: <https://cayman.loopnews.com/content/turks-and-caicos-intercepts-sixth-vessel-haitians-2023-0> |
| US Virgin Islands | Probable | M | The U.S. Virgin Islands have implemented a robust ASF prevention strategy in partnership with the USDA. Measures include a federal Protection Zone, strict import restrictions, routine swine farm testing, increased training to farmers, and heightened inspections at ports. This initiative included Customs and Border Protection to increase inspections of passengers, boats, aircraft, and cargo leaving/entering the USVI.  Haitian immigrants are using the porous borders of the British Virgin Islands to enter USVI through sea vessels . Due to the United States’ Homeland Security Temporary Protected Status Program, Haitian nationals have temporary work and travel authorization and relief from deportation.  There are other reports of immigrants being detained or charged with smuggling immigrants into the USVI. Imports of meat are generally prohibited, but there are reports of meat products bypassing customs enforcement. | | | Virgin Islands Department of Agriculture. The Virgin Islands Department of Agriculture continues disease surveillance of all swine and chicken avian farms in the territory [Internet]. U.S. Virgin Islands: DOA VI; 2024. [cited 2024 Nov-Dec]. Available from: <https://doa.vi.gov/the-virgin-islands-department-of-agriculture-continues-disease-surveillance-of-all-swine-and-chicken-avian-farms-in-the-territory/>  Euro Meat News. Federal order issued to protect Puerto Rico from ASF [Internet]. 2023. [cited 2024 Nov-Dec]. Available from: <https://www.euromeatnews.com/Article-Federal-order-issued-to-protect-Puerto-Rico-from-ASF/4962>  Virgin Islands Department of Agriculture. African swine fever [Internet]. U.S. Virgin Islands: DOA VI; 2024. [cited 2024 Nov-Dec]. Available from: <https://doa.vi.gov/asf/>  World Organisation for Animal Health. ASF in the Caribbean [Internet]. 2021. [cited 2024 Nov-Dec]. Available from: <https://www.woah.org/app/uploads/2021/10/2021-10-usa-asf-pz-uscaribbean.pdf>  United States Department of Agriculture. African swine fever part 2: Safeguarding the future [Internet]. 2022 Oct 14. [cited 2024 Nov-Dec]. Available from: <https://www.usda.gov/media/blog/2022/10/14/african-swine-fever-part-2-safeguarding-future>  United States Department of Agriculture. USDA APHIS bulletin [Internet]. 2024. [cited 2024 Nov-Dec]. Available from: <https://content.govdelivery.com/accounts/USDAAPHIS/bulletins/3741a70>  Virgin Islands Department of Agriculture. Swine avian newsletter [Internet]. 2022 Apr 11. [cited 2024 Nov-Dec]. Available from: <https://doa.vi.gov/wp-content/uploads/2022/04/SwineAvianNewsletter_Bradford_04.11.22.pdf>  Loop News Cayman. Turks and Caicos intercepts sixth vessel of Haitians in 2023 [Internet]. 2023. [cited 2024 Nov-Dec]. Available from: <https://cayman.loopnews.com/content/turks-and-caicos-intercepts-sixth-vessel-haitians-2023-0>  Financial Investigation Agency BVI. Migrant smuggling: Extent and impact in the Virgin Islands [Internet]. 2024. [cited 2024 Nov-Dec]. Available from: <https://fiabvi.vg/Portals/0/DNNGalleryPro/uploads/2024/10/3/MigrantSmuggling-ExtentandImpactintheVIforPublicUse(FINAL).pdf>  Tripadvisor. Bringing food to St. Thomas, U.S. Virgin Islands [Internet]. 2024. [cited 2024 Nov-Dec]. Available from: <https://www.tripadvisor.com/ShowTopic-g147404-i172-k14790901-Bringing_food-St_Thomas_U_S_Virgin_Islands.html>  VisaHQ. U.S. Virgin Islands customs regulations [Internet]. 2024. [cited 2024 Nov-Dec]. Available from: <https://www.visahq.com/us-virgin-islands/customs/>  International Moving. Moving to the U.S. Virgin Islands [Internet]. 2024. [cited 2024 Nov-Dec]. Available from: <https://internationalmoving.com/moving-to-usvirginislands/>  U.S. Customs and Border Protection. Migrant smuggling cases [Internet]. 2024. [cited 2024 Nov-Dec]. Available from: <https://www.cbp.gov/node/382902>  St. Thomas Source. Two BVI men attempt to smuggle aliens into USVI [Internet]. 2023 Sep 1. [cited 2024 Nov-Dec]. Available from: <https://stthomassource.com/content/2023/09/01/two-bvi-men-attempt-to-smuggle-aliens-into-usvi/> |
| Venezuela | Unknown, Probable | L | The country's economic collapse, poor infrastructure, and government corruption contribute to weak enforcement of biosecurity measures. The net outflow of migrants, reducing the likelihood of ASF-carrying imports through human travel. While customs prohibit meat imports, moderate to high numbers of informal pork products have been seized. Additionally, the presence of 94 aid organizations and ongoing humanitarian crises further complicate enforcement and monitoring efforts. | | | Associated Press. Human rights concerns in Venezuela and Haiti [Internet]. 2024. [cited 2024 Nov-Dec]. Available from: <https://apnews.com/article/human-rights-venezuela-haiti-refugees-068a54a18ddbab713cf5d969ba27ae30>  Council on Foreign Relations. Instability in Venezuela: Global conflict tracker [Internet]. 2024. [cited 2024 Nov-Dec]. Available from: <https://www.cfr.org/global-conflict-tracker/conflict/instability-venezuela>  Migration Policy Institute. Haitian migration through the Americas [Internet]. 2024. [cited 2024 Nov-Dec]. Available from: <https://www.migrationpolicy.org/article/haitian-migration-through-americas>  United Nations Office for the Coordination of Humanitarian Affairs. Humanitarian response plan for Venezuela [Internet]. 2024. [cited 2024 Nov-Dec]. Available from: <https://humanitarianaction.info/plan/1163?bs=eyJibG9jay0xNTRiYjNkMC00YmU1LTQ4ODItOWM1MC1kMTA2YzUxODIzMWEiOnsidGFyZ2V0IjoyfX0%3D>  United Nations High Commissioner for Refugees. Venezuela situation report [Internet]. 2024. [cited 2024 Nov-Dec]. Available from: <https://reporting.unhcr.org/operational/situations/venezuela-situation>  World Baggage Network. Customs regulations for Venezuela [Internet]. 2024. [cited 2024 Nov-Dec]. Available from: <https://www.worldbaggagenetwork.com/kb/venezuela/2-customs-regulations-for-your-trip-to-venezuela/> |
