# Supplementary material for: African swine fever incursion risks in Latin America and the Caribbean: informal and legal import pathways
Source: Front Vet Sci. 2025 Apr 1;12:1587131. doi: 10.3389/fvets.2025.1587131 (PMC11996821; doi:10.3389/fvets.2025.1587131)
Supplement: Supplementary file 2 [file Table_2.docx]

Supplemental Table 2: Risk factors and references for targeted territories in the legal imports pathway

|  |  | |  |  |
| --- | --- | --- | --- | --- |
| References for all countries: | Data on animal imports and exports by country and region, accessed from the World Integrated Trade Solution (WITS), World Bank. [Accessed Oct 2024-Dec 2024]  Data on live pork and pork products (HS 010391, 010310, 010392, 020311, 020319, 020322, 020312, 020321, 020329, 020630, 020649, 020641, 021011, 021019, 021012, 020900), accessed from the World Integrated Trade Solution (WITS), World Bank. [Accessed Oct 2024-Dec 2024]  Organismo Internacional Regional de Sanidad Agropecuaria. Análisis de riesgo sobre la probabilidad de ingreso, establecimiento y diseminación del virus de la peste porcina africana en la porcicultura de los países de la región del OIRSA  [Internet]. 2020 [cited Nov-Dec 2024]. Available from: https://web.oirsa.org/analisis-de-riesgo-sobre-la-probabilidad-de-ingreso-establecimiento-y-diseminacion-del-virus-de-la-peste-porcina-africana-en-la-porcicultura-de-los-paises-de-la-region-del-oirsa/  Rozstalnyy, A., Roche, X., TagoPacheco, D., Kamata, A., BeltranAlcrudo, D., Khomenko, S., Lockhart, C., Urdaz, J., Gioia, G., Gonzalez Serrano, A., VonDobschuetz, S., Dhingra, M., & Sumption, K. 2022. Qualitative risk assessment for African swine fever virus introduction – Caribbean, South, Central and North Americas. FAO Animal Production and Health Papers, No. 186. Rome. <https://doi.org/10.4060/cb8748en> | | | |
| Territory | **Level of Risk** | **Level of Certainty** | **Risk Factors** | **Sources** |
| Anguilla | Unknown, Unlikely | L | Biosecurity and surveillance measures for imported meat and animals  Historical (possibly still active) ban on pork importation due to ASF  No reports of pork or pig imports since 2017 to WITS | African swine fever in the Caribbean region. [Internet]. The Anguillian, 2021. [cited Nov-Dec, 2024]. Available from <https://theanguillian.com/2021/08/african-swine-fever-in-the-caribbean-region/3>  Anguilla. [Internet]. Observatory of Economic Complexity. [cited Nov-Dec 2024]. Available from <https://oec.world/en/profile/country/aia>  Livestock import permit. [Internet]. Ministry of economic development, investment, commerce, information technology and natural resources. [cited Nov-Dec 2024]. Available from <https://gov.ai/document/nationalresources/Import%20Permit-%20Livestock%20(eg%20Birds,%20Pig,%20Sheep,%20Goats)%20-%20(other%20pets%20excluding%20Cat%20&%20Dog).pdf> |
| Antigua and Barbuda | Unknown, Unlikely | L | Imports from ASF-free countries  Ban on pork product imports from Dominican Republic | Williams, O. Antigua and Barbuda avoiding swine flu amid nearby outbreak. [Internet]. Antigua Observer, 2021. [cited Nov-Dec 2024]. Available from <https://antiguaobserver.com/antigua-and-barbuda-avoiding-swine-flu-amid-nearby-outbreak/> |
| Bahamas | Probable | M | Biosecurity and surveillance measures for imported meat and animals  Allows import from countries/regions not ASF-free with veterinary certificate per WOAH guidelines | Ellis. A. Americas: Regional surveys on African swine fever risk and preparedness. [Internet]. World Organization for Animal Health, 2019. [cited Nov-Dec 2024]. Available from <https://rr-americas.woah.org/app/uploads/2020/01/cfia_acia-12196869-v1-gf-tads_asf_americas_2019_survey_analysis1.pdf>  Trade Facilitation Unit – Pork. [Internet]. Bahamas Agricultural Health Food Safety Authority. [cited Nov-Dec 2024]. Available from <https://bahfsabahamas.com/> |
| Barbados | Unlikely | M | Biosecurity and surveillance measures for imported meat and animals  Imports from ASF-free countries | Agriculture Ministry has plan to keep pig stock safe from African Swine Fever. [Internet]. Barbados Today, 2021. [cited Nov-Dec 2024]. Available from <https://barbadostoday.bb/2021/08/28/agriculture-ministry-has-plan-to-keep-pig-stock-safe-from-african-swine-fever/amp/>  Barbados. [Internet]. USDA FSIS, 2024. [cited Nov-Dec 2024]. Available from <https://www.fsis.usda.gov/inspection/import-export/import-export-library/barbados#:~:text=Fresh/frozen%20and%20Cooked%20Pork,on%20the%20AMS'%20Web%20site>.  Braithwaite, S. Pork imports surge ahead of World Cup. [Internet]. Barbados Today, 2024. [cited Nov-Dec 2024]. Available from <https://barbadostoday.bb/2024/04/17/pork-imports-surge-ahead-of-world-cup/>  Bringing animals into Barbados. [Internet]. Ministry of Agriculture. [cited Nov-Dec 2024]. Available from <https://agriculture.gov.bb/Departments/Veterinary-Services/Bringing-Animals-into-Barbados/#:~:text=Animals%20and%20birds%20may%20be,and%20when%20it%20enters%20Barbados>  Ellis. A. Americas: Regional surveys on African swine fever risk and preparedness. [Internet]. World Organization for Animal Health, 2019. [cited Nov-Dec 2024]. Available from <https://rr-americas.woah.org/app/uploads/2020/01/cfia_acia-12196869-v1-gf-tads_asf_americas_2019_survey_analysis1.pdf> |
| Belize | Unknown, Probable | L | Biosecurity and surveillance measures for imported meat and animals  Ban on imports of pigs and pork products from ASF-affected countries  Bedding and fodder disinfected or destroyed on entry  Reliance on regional support – in-country surveillance proactive but still developing | Belize – Country Commercial Guide: Trade Barriers. [Internet]. International Trade Administration, 2024. [cited Nov-Dec 2024]. Available from <https://www.trade.gov/country-commercial-guides/belize-trade-barriers>  Import Guidelines. [Internet]. Belize Agricultural Health Authority. [cited Nov-Dec 2024]. Available from <https://baha.org.bz/departments/animal-health/import-guidelines/>  Rubén Morales Iglesias. Belize Pig Council makes donation to Agricultural authorities for surveillance, monitoring activities in regards to African Swine fever. [Internet]. Breaking Belize News, 2021. [cited Nov-Dec 2024]. Available from: <https://www.breakingbelizenews.com/2021/08/06/belize-pig-council-makes-donation-to-agricultural-authorities-for-surveillance-monitoring-activities-in-regards-to-african-swine-fever/>  Training on African Swine Fever. [Internet]. Ministry of Agriculture, Food Security, and Enterprises, 2022. [cited Nov-Dec 2024]. Available from: <https://www.agriculture.gov.bz/training-on-african-swine-fever/> |
| Bermuda | Unlikely | M | Imports from ASF-free countries  No live animal quarantine facilities  Apparently no pigs in Bermuda, pigs not mentioned in any animal import listings | Prohibited and restricted goods. [Internet]. Government of Bermuda. [cited Nov-Dec 2024]. Available from <https://www.gov.bm/prohibited-and-restricted-goods>  Stevenson, C. No more pig farming in Bermuda? [Internet]. Bermuda Broadcasting Company, 2024. [cited Nov-Dec 2024]. Available from <https://www.facebook.com/bermudabroadcasting/videos/1409021416644079/> |
| Bonaire | Unknown, Unlikely | L | Ban on imports from DR | Bonaire. [Internet]. USDA FSIS, 2023. [cited Nov-Dec 2024]. Available from <https://www.fsis.usda.gov/inspection/import-export/import-export-library/bonaire>  Bonaire government issues import ban on meat from Dominican Republic. [Internet]. BES reporter, 2021. [cited Nov-Dec 2024]. Available from [bes-reporter.com/bonaire-government-issues-import-ban-on-meat-from-dominican-republic](https://bes-reporter.com/bonaire-government-issues-import-ban-on-meat-from-dominican-republic/#:~:text=Bonaire%20Government%20issues%20Import%20Ban%20on%20meat%20from%20Dominican%20Republic,-reporter&text=Kralendijk%20%E2%80%93%20It%20is%20temporarily%20prohibited,found%20in%20the%20Dominican%20Republic.) |
| British Virgin Islands | Unlikely | M | Biosecurity and surveillance measures for imported meat and animals  Imports from ASF-free countries | Conditions for importation of pigs. [Internet]. Government of the British Virgin Islands Veterinary Division. [cited Nov-Dec 2024]. Available from https://www.bvi.gov.vg/sites/default/files/Conditions%20for%20importation%20of%20pigs.pdf  Import conditions beef & pork. [Internet]. Government of the British Virgin Islands Veterinary Division. [cited Nov-Dec 2024]. Available from <https://bvi.gov.vg/sites/default/files/meat_import_conditions.pdf>  Import health requirements of the British Virgin Islands for swine exported from the United States. [Internet]. USDA APHIS, 2001. [cited Nov-Dec 2024]. Available from <https://www.aphis.usda.gov/sites/default/files/vg_po.pdf> |
| Cayman Islands | Unlikely | M | Biosecurity and surveillance measures for imported meat and animals  Imports from ASF-free countries  Ban on imports of pigs and pork products from ASF-affected countries | Cayman Islands. [Internet]. USDA FSIS, 2024. [cited Nov-Dec 2024]. Available from <https://www.fsis.usda.gov/inspection/import-export/import-export-library/cayman-islands#:~:text=Other%20Requirements,bears%20the%20USDA%20inspection%20legend>.  Conditions governing the importation of pigs into the Cayman Islands. [Internet]. Cayman Islands Department of Agriculture. [cited Nov-Dec 2024]. Available from <https://cnslocallife.com/wp-content/uploads/2016/08/Pig-Import-Conditions-Rev-01-2015.pdf>  Conditions governing the importation of meat and meat products (excluding game meat) into the Cayman Islands. [Internet]. Cayman Islands Department of Agriculture. [cited Nov-Dec 2024]. Available from <https://doa.gov.ky/wp-content/uploads/2024/10/OF-IMP-12-IMPORTATION-OF-MEAT-AND-MEAT-PRODUCTS-Revised-2024.pdf> |
| Colombia | Unlikely | H | Extensive biosecurity and surveillance measures for imported meat and animals  Imports from ASF-free countries | Colombia: caen las importaciones de carne de cerdo por quinto mes consecutive. [Internet]. Pig333, 2023. [cited Nov-Dec 2024]. Available from <https://www.3tres3.com/latam/ultima-hora/colombia-caen-las-importaciones-de-carne-de-cerdo-por-quinto-mes_15960/>  Colombia's import restrictions due to African swine fever (ID 580). [Internet]. World Trade Organization. [cited Nov-Dec 2024]. Available from <https://tradeconcerns.wto.org/en/stcs/details?imsId=580&domainId=SPS>  Importación Pecuaria. [Internet]. Instituto Colombiano Agropecuario. [cited Nov-Dec 2024]. Available from <https://www.ica.gov.co/importacion-y-exportacion/procedimientos-importacion>  México y Colombia toman medidas frente a la confirmación de PPA en República Dominicana. [Internet]. Pig 333, 2021. [cited Nov-Dec 2024]. Available from <https://www.3tres3.com/latam/ultima-hora/mexico-y-colombia-toman-medidas-ante-la-ppa-en-republica-dominicana_13375/>  Workman D. Pork Imports by Country [Internet]. Worlds Top Exports. [cited Nov-Dec 2024]. Available from: https://www.worldstopexports.com/international-markets-for-imported-pork-by-country/ |
| Costa Rica | Unlikely | M | Biosecurity and surveillance measures for imported meat and animals  Passive and active surveillance at farms and slaughterhouses  Imports from ASF-free countries | Costa Rica refuerza controles para evitar ingreso de peste porcina africana. [Internet]. Swissinfo.ch, 2021. [cited Nov-Dec 2024]. Available from <https://www.swissinfo.ch/spa/costa-rica-refuerza-controles-para-evitar-ingreso-de-peste-porcina-africana/46831630>  Costa Rica: Slaughter, pork imports, and production costs in Jan-Apr. [Internet]. Pig333, 2024. [cited Nov-Dec 2024]. Available from <https://www.pig333.com/latest_swine_news/costa-ricas-hog-slaughter-pork-imports-and-production-costs_20406/>  Guias al usuario. [Internet]. Servicio Nacional de Salud Animal. [cited Nov-Dec 2024]. Available from <https://www.senasa.go.cr/tramites-y-servicios/guias-al-usuario>  Main imported products in Costa Rica in 2023, by value. [Internet]. Statista, 2024. [cited Nov-Dec 2024]. Available from <https://www.statista.com/statistics/1191408/products-imported-to-costa-rica/>  N° SENASA-DCA-R0029-2021. Ministerio de Agricultura y Ganadería de Costa Rica, 2021. [cited Nov-Dec 2024]. Available from <file:///C:/Users/schu4994/Downloads/SENASA-DG-R0029-2021%20Resolucion%20cierre%20temporal%20PPA.pdf>  Pork imports in Costa Rica – Market size and demand based on trade import data. [Internet]. Volza. [cited Nov-Dec 2024]. Available from <https://www.volza.com/p/pork/import/import-in-costa-rica/> |
| Cuba | Probable | M | Imports from Russia, a country with current ASF outbreaks; minimal information on biosecurity measures put in place  Ban on imports from Haiti and DR | Alertan en Cuba sobre presencia de peste porcina africana en República Dominicana. [Internet]. OnCuba, 2021. [cited Nov-Dec 2024]. Available from <https://oncubanews.com/cuba/alertan-en-cuba-sobre-presencia-de-peste-porcina-africana-en-republica-dominicana/>  Cuba. [Internet]. USDA FSIS, 2024. [cited Nov-Dec 2024]. Available from <https://www.fsis.usda.gov/inspection/import-export/import-export-library/cuba>  Cuba endorses 30 federal inspection type plants to export pork, beef and poultry. [Internet]. Government of Mexico, 2022. [cited Nov-Dec 2024]. Available from <https://www.gob.mx/senasica/documentos/cuba-endorses-30-federal-inspection-type-plants-to-export-pork-beef-and-poultry>  Empresas rusas podrán vender carne de pollo, cerdo y vacuno a Cuba. [Internet]. En Alimentos, 2024. [cited Nov-Dec2024]. Available from <https://enalimentos.lat/noticias/10084-empresas-rusas-podran-vender-carne-de-pollo-cerdo-y-vacuno-a-cuba.html>  Russian companies obtain authorization to export meat and dairy products to Cuba. [Internet]. CiberCuba, 2024. [cited Nov-Dec 2024]. Available from <https://en.cibercuba.com/noticias/2024-10-31-u1-e197721-s27061-nid291186-empresas-rusas-obtienen-autorizacion-exportar-carnes> |
| Curaçao | Unlikely | M | Biosecurity and surveillance measures for imported meat and animals | Animal health. [Internet]. Ministerie van Gezondheid, Milieu en Natuur UO Veterinaire Zaken. [cited Nov-Dec 2024]. Available from <https://uovz.cw/organization/animal-health/>  Import permission for products of animal origin. [Internet]. Ministerie van Gezondheid, Milieu en Natuur UO Veterinaire Zaken. [cited Nov-Dec 2024]. Available from <https://uovz.cw/import/import-of-products-of-animal-origin/> |
| Dominica | Unlikely | M | Biosecurity and surveillance measures for imported meat and animals  Imports from ASF-free countries | Dominica. [Internet]. USDA FSIS, 2024. [cited Nov-Dec 2024]. Available from <https://www.fsis.usda.gov/inspection/import-export/import-export-library/dominica>  Livestock development & veterinary unit. [Internet]. Government of the Commonwealth of Dominica, Division of Agriculture. [cited Nov-Dec 2024]. Available from <https://divisionofagriculture.gov.dm/units/livestock-development-veterinary-unit> |
| El Salvador | Unlikely | M | Biosecurity and surveillance measures for imported meat and animals  Ban on imports of pigs and pork products from ASF-affected countries  Disinfection at points of entry for incoming containers from ASF-affected countries | Cuarentena animal. [Internet]. Ministerio de Agricultura y Ganadería. [cited Nov-Dec 2024]. Available from <https://www.mag.gob.sv/servicios/cuarentena-animal-y-registro-veterinario/>  El Salvador fortalice medidas de prevención ante peste porcina africana (PPA). [Internet]. Organismo Internacional Regional de Sanidad Agropecuaria, 2019. [cited Nov-Dec 2024]. Available from <https://web.oirsa.org/en/el-salvador-fortalece-medidas-de-prevencion-ante-peste-porcina-africana-ppa/>  France-Presse, A. El Salvador bans pork imports over African swine fever fears. [Internet]. NDTV World, 2021. [cited Nov-Dec 2024]. Available from <https://www.ndtv.com/world-news/el-salvador-bans-pork-imports-over-african-swine-fever-fears-2500533>  Inspección en origen. [Internet]. Ministerio de Agricultura y Ganadería. [cited Nov-Dec 2024]. Available from <https://www.mag.gob.sv/inspeccion-en-origen/>  Main imported products in El Salvador in 2023, by value. [Internet]. Statista, 2024. [cited Nov-Dec 2024]. Available from <https://www-statista-com.ezp2.lib.umn.edu/statistics/1191371/products-imported-to-el-salvador/> |
| Grenada | Unknown, Unlikely | L | Imports from ASF-free countries | Grenada. [Internet]. USDA FSIS, 2024. [cited Nov-Dec 2024]. Available from <https://www.fsis.usda.gov/inspection/import-export/import-export-library/grenada> |
| Guadeloupe | Unknown, Unlikely | L | Participation in capacity building projects  Majority of trade with France | Exporter guide. [Internet]. USDA Foreign Agriculture Service, 2024. [cited Nov-Dec 2024]. Available from https://apps.fas.usda.gov/newgainapi/api/Report/DownloadReportByFileName?  fileName=Exporter%20Guide_Miami%20ATO_%20Basin_C12023-0003.pdf  France. [Internet]. USDA FSIS, 2024. [cited Nov-Dec 2024]. Available from <https://www.fsis.usda.gov/inspection/import-export/import-export-library/grenada>  One health approach to reduce health risks in the Caribbean – AUSCAR. [Internet]. CaribVET. [cited Nov-Dec 2024]. Available from <https://www.caribvet.net/projects/auscar> |
| Guatemala | Unlikely | M | Biosecurity and surveillance measures for imported meat and animals  Imports from ASF-free countries | Guatemala endurece vigilancia fronteriza para evitar ingreso de peste porcina. [Internet]. Swissinfo.ch, 2022. [cited Nov-Dec 2024]. Available from: <https://www.swissinfo.ch/spa/guatemala-endurece-vigilancia-fronteriza-para-evitar-ingreso-de-peste-porcina/47260712>  Importaciones. [Internet]. Ministerio de Agricultura, Ganadería y Alimentación. [cited Nov-Dec 2024]. Available from: <https://www.maga.gob.gt/sitios/visar/importaciones/#1729103044524-ac664c3d-318c> |
| Guyana | Unlikely | M | Extensive biosecurity and surveillance measures for imported meat and animals  Imports from ASF-free countries  Historical (possibly still active) ban on pork importation due to ASF | ‘African Swine Fever’ outbreak triggers immediate ban on importation of pork products. [Internet]. Kaieteur News, 2021. [cited Nov-Dec 2024]. Available from <https://www.kaieteurnewsonline.com/2021/11/16/african-swine-fever-outbreak-triggers-immediate-ban-on-importation-of-pork/>  Procedure for the importation of animals into Guyana. [Internet]. Ministry of Agriculture. [cited Nov-Dec 2024]. <https://agriculture.gov.gy/2016/06/27/procedure-for-the-importation-of-animals-into-guyana/>  Regulations made under Animal Health Act 2011. [Internet]. Ministry of Agriculture, 2016. [cited Nov-Dec 2024]. Available from <https://agriculture.gov.gy/wp-content/uploads/2016/02/REGULATIONS.pdf>  Over $977 M to stimulate livestock industry in 2022 – Minister Mustapha. [Internet]. Ministry of Agriculture, 2022. [cited Nov-Dec 2024]. Available from <https://agriculture.gov.gy/2022/02/03/over-977-m-to-stimulate-livestock-industry-in-2022-minister-mustapha/>  Steps taken to better equip GLDA’s Epidemiology Unit. [Internet]. Department of Public Information, 2021. [cited Nov-Dec 2024]. Available from <https://dpi.gov.gy/steps-taken-to-better-equip-gldas-epidemiology-unit/> |
| Honduras | Unlikely | M | Biosecurity and surveillance measures for imported meat and animals  Imports from ASF-free countries | Honduras crea cerco contra la Peste Porcina Africana. [Internet]. La Tribuna. [cited Nov-Dec 2024]. Available from <https://www.latribuna.hn/2024/09/04/honduras-crea-cerco-contra-la-peste-porcina-africana/>  Honduras – country commercial guide: Import requirements and documentation. [Internet]. International Trade Administration. [cited Nov-Dec 2024]. Available from <https://www.trade.gov/country-commercial-guides/honduras-import-requirements-and-documentation>  Data on imports accessed from SENASA. [cited Nov-Dec 2024]. Available from <http://importaciones.senasa.gob.hn/#/consultar-requisito>  Main imported products in Honduras in 2023, by value. [Internet]. Statista. [cited Nov-Dec 2024]. Available from <https://www-statista-com.ezp3.lib.umn.edu/statistics/1191197/products-imported-to-honduras/>  Pig Meat in Honduras. [Internet]. Observatory of Economic Complexity. [cited Nov-Dec 2024]. Available from <https://oec.world/en/profile/bilateral-product/pig-meat/reporter/hnd>  Vigilancia de Enfermedades Porcinas. [Internet]. SENASA. [cited Nov-Dec 2024]. Available from <https://senasa.gob.hn/vigilancia-de-enfermedades-en-equinos-2/> |
| Jamaica | Unlikely | M | Extensive biosecurity and surveillance measures for imported meat and animals  Intensified surveillance and early detection activities  Limited importation of pork | Agriculture Ministry now able to investigate and diagnose African swine fever. [Internet]. Ministry of Agriculture, Fisheries, and Mining, 2021. [cited Nov-Dec 2024]. Available from <https://www.moa.gov.jm/content/agriculture-ministry-now-able-investigate-and-diagnose-african-swine-fever>  Ellis. A. Americas: Regional surveys on African swine fever risk and preparedness. [Internet]. World Organization for Animal Health, 2019. [cited Nov-Dec 2024]. Available from <https://rr-americas.woah.org/app/uploads/2020/01/cfia_acia-12196869-v1-gf-tads_asf_americas_2019_survey_analysis1.pdf>  Jamaica. [Internet]. USDA FSIS, 2024. [cited Nov-Dec 2024]. Available from <https://www.fsis.usda.gov/inspection/import-export/import-export-library/jamaica>  Jones, R. Hogwash! - US wants cut of local pork market but Jamaican pig farmers say no. [Internet]. Jamaica Gleaner, 2016. [cited Nov-Dec 2024]. Available from <https://jamaica-gleaner.com/article/news/20160522/hogwash-us-wants-cut-local-pork-market-jamaican-pig-farmers-say-no> |
| Martinique | Unknown, Unlikely | L | Majority of trade with France | Exporter guide. [Internet]. USDA Foreign Agriculture Service, 2024. [cited Nov-Dec 2024]. Available from https://apps.fas.usda.gov/newgainapi/api/Report/DownloadReportByFileName?  fileName=Exporter%20Guide_Miami%20ATO_%20Basin_C12023-0003.pdf  France. [Internet]. USDA FSIS, 2024. [cited Nov-Dec 2024]. Available from <https://www.fsis.usda.gov/inspection/import-export/import-export-library/grenada> |
| Mexico | Unlikely | M | Participated in biosecurity strengthening exercises  Extensive biosecurity and surveillance measures for imported meat and animals | Canada, Mexico, and US unite to keep North America ASF-free. [Internet]. National Hog Farmer; 2019 [cited Nov-Dec 2024]. Available from: https://www.nationalhogfarmer.com/hog-health/canada-mexico-and-u-s-unite-to-keep-north-america-asf-free  Export Animal Products to Mexico [Internet]. [cited Nov-Dec 2024]. Available from: https://www.aphis.usda.gov/animal-product-export/export-animal-products-mexico  Mexico: Actions and measures to prevent the entry of ASF [Internet]. Pig333; 2019 [cited Nov-Dec 2024]. Available from: https://www.pig333.com/latest_swine_news/mexico-actions-and-measures-to-prevent-the-entry-of-asf_14917/  Mexico activates the “Integral Biosecurity” plan, facing the African Swine Fever (ASF) threat. [Internet]. Gobierna de Mexico [cited Nov-Dec 2024]. Available from: https://www.gob.mx/cms/uploads/attachment/file/659710/Mexico_activates_  the_Integral_Biosecurity_plan__facing_the_African_Swine_Fever_ASF_threat.pdf  Mexico strengthens the surveillance in borders and airports to avoid the entrance of ASF [Internet]. Pig333; 2019 [cited Nov-Dec 2024]. Available from: https://www.pig333.com/latest_swine_news/mexico-strengthens-border-and-airport-surveillance-to-avoid-asf_14533/  Mexico: Integral biosecurity to prevent the entry of ASF [Internet]. Pig333; 2016 [cited Nov-Dec 2024]. Available from: https://www.pig333.com/latest_swine_news/mexico-integral-biosecurity-to-prevent-the-entry-of-asf_15157/  Proyecto Regional de Erradicación de Peste Porcina Clásica (PPC). [Internet]. Organismo Internacional Regional de Sanidad Agropecuaria; 2023 [cited Nov-Dec 2024]. Available from: https://web.oirsa.org/programa-regional-de-sanidad-porcina/  Six-monthly report on the notification of listed diseases, infections and infestations: Second semester 2023 - Mexico - Terrestrial. World Organisation for Animal Health; 2023. Available from: https://wahis.woah.org/#/smr-review/166165?reportInfoId=93777&fromPage=smr-management-url  We do not lower our guard against ASF [Internet]. Gobierna de Mexico; 2022 [cited Nov-Dec 2024]. Available from: <https://www.gob.mx/senasica/documentos/we-do-not-lower-our-guard-against-asf>  Workman D. Pork Imports by Country [Internet]. Worlds Top Exports. [cited Nov-Dec 2024]. Available from: https://www.worldstopexports.com/international-markets-for-imported-pork-by-country/ |
| Montserrat | Unknown, Unlikely | L | Imports from ASF-free countries |  |
| Nicaragua | Unlikely | M | Extensive biosecurity and surveillance measures for imported meat and animals  Imports from ASF-free countries | Evan Mangino. FAIRS Country Report Annual. [Internet]. USDA Foreign Agricultural Service, 2024. [cited Nov-Dec 2024]. Available from https://apps.fas.usda.gov/newgainapi/api/Report/DownloadReportByFileName?fileName=  FAIRS%20Country%20Report%20Annual_Managua_Nicaragua_NU2024-0008  Export live animals to Nicaragua. [Internet]. USDA, 2024. [cited Nov-Dec 2024]. Available from <https://www.aphis.usda.gov/live-animal-export/export-live-animals-nicaragua>  Medidas sanitarias para la prevención de la introducción de la peste porcina africana: resolución ejecutiva N°. 067-2019. [Internet]. Legislación de Nicaragua, 2019. [cited Nov-Dec 2024]. Available from http://legislacion.asamblea.gob.ni/Normaweb.nsf/3133c0d121ea3897062568a1005e0f89/  6d43fff3e305b79d062584a500520b26?OpenDocument |
| Panama | Unlikely | H | Extensive biosecurity and surveillance measures for imported meat and animals  Documented collaboration between public and private sector for ASF prevention  Imports from ASF-free countries | de Escobar, C. Normas de la OMSA, comercio y Peste Porcina Africana (PPA): Panama. [Internet]. República de Panamá, 2022. [cited Nov-Dec 2024]. Available from <https://rr-americas.woah.org/app/uploads/2022/11/0204d-esp_experiencia-panama.pdf>  Dirección Nacional de Salud Animal. [Internet]. Ministerio de Desarrollo Agropecuario. [cited Nov-Dec 2024]. Available from <https://mida.gob.pa/direcciones/direccion-nacional-de-salud-animal/?csrt=12651289305386630214#funciones>  Panama could restrict pork imports. [Internet]. Pig333, 2019. [cited Nov-Dec 2024]. Available from <https://www.pig333.com/latest_swine_news/panama-could-restrict-pork-imports_15017/>  Panama’s attempt to cap pork imports key test for FTA enforcement. [Internet]. National Hog Farmer, 2024. [cited Nov-Dec 2024]. Available from <https://www.nationalhogfarmer.com/market-news/panama-s-attempt-to-cap-pork-imports-key-test-for-fta-enforcement>  Panama Special Economic Zones. [Internet]. International Trade Administration. [cited Nov-Dec 2024]. Available from <https://www.trade.gov/market-intelligence/panama-special-economic-zones>  Pork imports in Panama – Market size and demand based on import trade data. [Internet]. Volza. [cited Nov-Dec 2024]. Available from <https://www.volza.com/p/pork/import/import-in-panama/>  Workman D. Pork Imports by Country [Internet]. World’s top exports. [cited Nov-Dec 2024]. Available from: <https://www.worldstopexports.com/international-markets-for-imported-pork-by-country/> |
| Puerto Rico | Unlikely | M | Extensive biosecurity and surveillance measures for imported meat and animals  Ban in imports of live swine  Imports pork products from ASF free countries  Protection Zone established by USDA APHIS | APHIS Celebrates African Swine Fever Preparedness and Prevention Accomplishments. [Internet]. United States Department of Agriculture Animal and Plant Health Inspection Service, 2023. [cited Nov-Dec 2024]. Available from <https://content.govdelivery.com/accounts/USDAAPHIS/bulletins/3741a70>  IndexBox. Exploring the top import markets for frozen pork cut. [Internet]. Global Trade, 2024. Available from <https://www.globaltrademag.com/exploring-the-top-import-markets-for-frozen-pork-cut/>  Self-declaration of the establishment of a Protection Zone for U.S. Territories in the Caribbean. [Internet]. World Organization for Animal Health, 2021. [cited Nov-Dec 2024]. Available from <https://www.woah.org/app/uploads/2021/10/2021-10-usa-asf-pz-uscaribbean.pdf>  State regulations for Puerto Rico. [Internet]. Government of Puerto Rico Department of Agriculture, 2023. [cited Nov-Dec 2024]. Available from <https://www.aphis.usda.gov/media/document/1546/file> |
| Saba | Unknown, Unlikely | L |  | Saba. [Internet]. USDA FSIS, 2023. [cited Nov-Dec 2024]. Available from <https://www.fsis.usda.gov/inspection/import-export/import-export-library/saba> |
| Saint Barthelemy | Unknown, Unlikely | L | No preventive actions publicly available |  |
| Saint Lucia | Unlikely | M | Biosecurity and surveillance measures for imported meat and animals  Imports from ASF-free countries  Developing swine industry to reduce imports | Ellis. A. Americas: Regional surveys on African swine fever risk and preparedness. [Internet]. World Organization for Animal Health, 2019. [cited Nov-Dec 2024]. Available from <https://rr-americas.woah.org/app/uploads/2020/01/cfia_acia-12196869-v1-gf-tads_asf_americas_2019_survey_analysis1.pdf>  Quarantine and regulatory services. [Internet]. Saint Lucia Ministry of Agriculture. [cited Nov-Dec 2024]. Available from <https://moaslu.govt.lc/quarantine-and-regulatory-services/>  Saint Lucia. [Internet]. USDA FSIS, 2023. [cited Nov-Dec 2024]. Available from <https://www.fsis.usda.gov/inspection/import-export/import-export-library/st--lucia>  Saint Lucia sets sights on stepping up pork and ginger production. [Internet]. Food and Agriculture Organization, 2023. [cited Nov-Dec 2024]. Available from <https://www.fao.org/americas/news/news-detail/Saint-Lucia-sets-sights-on-stepping-up-pork-and-ginger-production-/en>  Veterinary & livestock services. [Internet]. Saint Lucia Ministry of Agriculture. [cited Nov-Dec 2024]. Available from <https://moaslu.govt.lc/veterinary-live-stock-services/> |
| Saint Martin | Unknown, Unlikely | L | Ban on imports from Dominican Republic | Continued ban on all pork products originating from the Dominican Republic due to positive African Swine Fever contaminated products. [Internet]. St. Martin News Network, 2022. [cited Nov-Dec 2024]. Available from <https://smn-news.com/index.php/st-maarten-st-martin-news/39650-continued-ban-on-all-pork-products-originating-from-the-dominican-republic-due-to-positive-african-swine-fever-contaminated-products.html>  Temporary ban on all pork products originating from the Dominican Republic due to the outbreak of African Swine Fever. [Internet]. St. Martin News Network, 2021. [cited Nov-Dec 2024]. Available from <https://smn-news.com/st-maarten-st-martin-news/38230-temporary-ban-on-all-pork-products-originating-from-the-dominican-republic-due-to-the-outbreak-of-african-swine-fever.html> |
| Sint Eustatius | Unknown, Unlikely | L |  |  |
| Sint Maarten | Unknown, Unlikely | L | Ban on pork imports from DR | [Internet]. Government of Sint Maarten (Facebook post), 2021. [cited Nov-Dec 2024]. Available from <https://www.facebook.com/SXMGOV/posts/-temporary-ban-on-all-pork-products-originating-from-the-dominican-republic-due-/2007793802725268>  Sint Maarten. [Internet]. USDA FSIS, 2021. [cited Nov-Dec 2024]. Available from <https://www.fsis.usda.gov/inspection/import-export/import-export-library/sint-maarten> |
| St Kitts and Nevis | Unknown, Unlikely | L | Biosecurity and surveillance measures for imported meat and animals  Imports from ASF-free countries | Dept. of agriculture informs SKN of re-emerging threat of African swine fever. [Internet]. Von Radio. [cited Nov-Dec 2024]. Available from <https://vonradio.com/dept-of-agriculture-informs-skn-of-re-emerging-threat-of-african-swine-fever/>  SKNISEditor. African swine flu bulletin. [Internet]. St. Kitts Nevis Information Service, 2023. [cited Nov-Dec 2024]. Available from <https://www.sknis.gov.kn/2023/12/20/african-swine-flu-bulletin/>  SKNISEditor. List of countries approved for imported meat products to St. Kitts. [Internet]. St. Kitts Nevis Information Service, 2023. [cited Nov-Dec 2024]. Available from <https://www.sknis.gov.kn/2023/12/21/list-of-countries-approved-for-imported-meat-products-to-st-kitts/>  St. Kitts and Nevis. [Internet]. USDA FSIS, 2021. [cited Nov-Dec 2024]. Available from <https://www.fsis.usda.gov/inspection/import-export/import-export-library/st--kitts-and-nevis>  Veterinary Import Permit for Swine. [Internet]. St. Christopher and Nevis Department of Agriculture. [cited Nov-Dec 2024]. Available from <https://skncustoms.com/Forms%20and%20Guides/VETERINARY%20IMPORT%20PERMIT%20FOR%20SWINE.pdf> |
| St. Vincent and the Grenadines | Unlikely | M | Biosecurity and surveillance measures for imported meat and animals  Imports from ASF-free countries | Animal health & production division introduction. [Internet]. The Government of Saint Vincent and the Grenadines Ministry of Agriculture, Forestry, Fisheries, Rural Transformation, Industry, and Labour. [cited Nov-Dec 2024]. Available from <https://agriculture.gov.vc/agriculture/index.php?option=com_content&view=article&id=23:animal-health-a-production-division-introduction&catid=104&Itemid=288>  Ellis. A. Americas: Regional surveys on African swine fever risk and preparedness. [Internet]. World Organization for Animal Health, 2019. [cited Nov-Dec 2024]. Available from <https://rr-americas.woah.org/app/uploads/2020/01/cfia_acia-12196869-v1-gf-tads_asf_americas_2019_survey_analysis1.pdf>  Import health requirements of St. Vincent for breeding swine exported from the United States. [Internet]. United States Department of Agriculture Animal and Plant Health Inspection Service, 2000. [cited Nov-Dec 2024]. Available from <https://www.aphis.usda.gov/sites/default/files/vc_po_br.pdf>  St. Vincent and the Grenadines. [Internet]. USDA FSIS, 2021. [cited Nov-Dec 2024]. Available from <https://www.fsis.usda.gov/inspection/import-export/import-export-library/st--vincent-and-grenadines>  SVG issues ‘RED ALERT’ for the African swine fever. [Internet]. T. Vincent Times, 2021. [cited Nov-Dec 2024]. Available from <https://www.stvincenttimes.com/svg-issues-red-alert-for-the-african-swine-fever/> |
| Suriname | Unlikely | M | Biosecurity and surveillance measures for imported meat and animals  Imports from ASF-free countries, no reports of pork or pig imports since 2017 to WITS | A quarterly update of key activities of the agricultural competitiveness program in Suriname. [Internet]. Agricultural Competitiveness Project, 2023. [cited 21 February 2025]. Available from <https://gov.sr/wp-content/uploads/2023/05/LVV-IDB-Newsletter-issue-01.pdf>  Export animal products to Suriname. [Internet]. USDA, 2024. [cited 21 February 2025]. Available from <https://www.aphis.usda.gov/animal-product-export/history/export-animal-products-suriname>  Food safety. [Internet]. Programme de Gestion Durable de la Faune Sauvage: Suriname. [cited Nov-Dec 2024]. Available from <https://www.swm-programme.info/fr/legal-hub/suriname/food-safety> |
| Trinidad and Tobago | Unlikely | M | Biosecurity and surveillance measures for imported meat and animals  Imports from ASF-free countries  Ban on imports of pigs and pork products from ASF-affected countries | African swine fever alert in the Caribbean region. [Internet]. Government of the Republic of Trinidad and Tobago Ministry of Agriculture, Land and Fisheries. [cited Nov-Dec]. Available from <https://agriculture.gov.tt/media-releases/african-swine-fever-alert-in-the-caribbean-region/>  *Animals (Diseases and Importation) Act 1997*. (Trinidad and Tobago) s 13.  Ellis. A. Americas: Regional surveys on African swine fever risk and preparedness. [Internet]. World Organization for Animal Health, 2019. [cited Nov-Dec 2024]. Available from <https://rr-americas.woah.org/app/uploads/2020/01/cfia_acia-12196869-v1-gf-tads_asf_americas_2019_survey_analysis1.pdf>  Trinidad and Tobago. [Internet]. USDA FSIS, 2021. [cited Nov-Dec 2024]. Available from <https://www.fsis.usda.gov/inspection/import-export/import-export-library/trinidad-and-tobago> |
| Turks and Caicos | Unknown, Unlikely | L | Minimal biosecurity and surveillance measures for imported meat and animals | Ellis. A. Americas: Regional surveys on African swine fever risk and preparedness. [Internet]. World Organization for Animal Health, 2019. [cited Nov-Dec 2024]. Available from <https://rr-americas.woah.org/app/uploads/2020/01/cfia_acia-12196869-v1-gf-tads_asf_americas_2019_survey_analysis1.pdf>  Prohibited and restricted goods. [Internet]. Turks and Caicos Islands Customs Department. [cited Nov-Dec 2024]. Available from <https://customs.gov.tc/webuploads/currdoc/Prohibited%20and%20restricted%20goods-Turks%20and%20Caicos.pdf>  Turks and Caicos. [Internet]. USDA FSIS, 2021. [cited Nov-Dec 2024]. Available from <https://www.fsis.usda.gov/inspection/import-export/import-export-library/turks-caicos-islands> |
| US Virgin Islands | Unlikely | M | Extensive biosecurity and surveillance measures for imported meat and animals  Protection Zone established by USDA APHIS | APHIS Celebrates African Swine Fever Preparedness and Prevention Accomplishments. [Internet]. United States Department of Agriculture Animal and Plant Health Inspection Service, 2023. [cited Nov-Dec 2024]. Available from <https://content.govdelivery.com/accounts/USDAAPHIS/bulletins/3741a70>  Self-declaration of the establishment of a Protection Zone for U.S. Territories in the Caribbean. [Internet]. World Organization for Animal Health, 2021. [cited Nov-Dec 2024]. Available from <https://www.woah.org/app/uploads/2021/10/2021-10-usa-asf-pz-uscaribbean.pdf>  State regulations for the Virgin Islands. [Internet]. Virgin Islands Department of Agriculture. [cited Nov-Dec 2024]. Available from [https://doa.vi.gov/animal-imports/](https://doa.vi.gov/animal-imports/#:~:text=All%20livestock%2C%20poultry%2C%20birds%2C%20and%20other%20animals%20must%20have,been%20found%20free%20of%20screwworms.) |
| Venezuela | Possible | M | Import of pork from ASF-affected country, with minimal published data on protective measures | Alerta por Venezuela: Senasa pidió información por la llegada de cerdos rusos. [Internet]. Agrofy News, 2020. [cited Nov-Dec 2024]. Available from <https://news.agrofy.com.ar/noticia/184996/alerta-venezuela-senasa-pidio-informacion-llegada-cerdos-rusos>  Venezuela: panorama general de la industria porcina. [Internet]. Pig333, 2024. [cited Nov-Dec 2024]. Available from <https://www.3tres3.com/latam/ultima-hora/venezuela-panorama-general-de-la-industria-porcina_17347/>  Russian pork in Venezuela worries vets in South America. [Internet]. Pig Progress, 2020. [cited Nov-Dec 2024]. Available from <https://www.pigprogress.net/world-of-pigs/russian-pork-in-venezuela-worries-vets-in-south-america/> |
